# The Effect of Factor VIII Deficiencies and Replacement and Bypass Therapies on Thrombus Formation under Venous Flow Conditions in Microfluidic and Computational Models

**DOI:** 10.1371/journal.pone.0078732

**Published:** 2013-11-13

**Authors:** Abimbola A. Onasoga-Jarvis, Karin Leiderman, Aaron L. Fogelson, Michael Wang, Marilyn J. Manco-Johnson, Jorge A. Di Paola, Keith B. Neeves

**Affiliations:** 1 Department of Chemical and Biological Engineering, Colorado School of Mines, Golden, Colorado, United States of America; 2 Applied Math Unit, School of Natural Sciences, University of California Merced, Merced, California, United States of America; 3 Department of Mathematics and Department of Bioengineering, University of Utah, Salt Lake City, Utah, United States of America; 4 Department of Pediatrics, Hemophilia and Thrombosis Center, University of Colorado Denver, Aurora, Colorado, United States of America; Emory University/Georgia Insititute of Technology, United States of America

## Abstract

Clinical evidence suggests that individuals with factor VIII (FVIII) deficiency (hemophilia A) are protected against venous thrombosis, but treatment with recombinant proteins can increase their risk for thrombosis. In this study we examined the dynamics of thrombus formation in individuals with hemophilia A and their response to replacement and bypass therapies under venous flow conditions. Fibrin and platelet accumulation were measured in microfluidic flow assays on a TF-rich surface at a shear rate of 100 s^−1^. Thrombin generation was calculated with a computational spatial-temporal model of thrombus formation. Mild FVIII deficiencies (5–30% normal levels) could support fibrin fiber formation, while severe (<1%) and moderate (1–5%) deficiencies could not. Based on these experimental observations, computational calculations estimate an average thrombin concentration of ∼10 nM is necessary to support fibrin formation under flow. There was no difference in fibrin formation between severe and moderate deficiencies, but platelet aggregate size was significantly larger for moderate deficiencies. Computational calculations estimate that the local thrombin concentration in moderate deficiencies is high enough to induce platelet activation (>1 nM), but too low to support fibrin formation (<10 nM). In the absence of platelets, fibrin formation was not supported even at normal FVIII levels, suggesting platelet adhesion is necessary for fibrin formation. Individuals treated by replacement therapy, recombinant FVIII, showed normalized fibrin formation. Individuals treated with bypass therapy, recombinant FVIIa, had a reduced lag time in fibrin formation, as well as elevated fibrin accumulation compared to healthy controls. Treatment of rFVIIa, but not rFVIII, resulted in significant changes in fibrin dynamics that could lead to a prothrombotic state.

## Introduction

Hemophilia A (HA) is an X-linked genetic disorder that results in deficiencies of coagulation factor VIII (FVIII). The primary clinical manifestation of FVIII deficiencies is bleeding in the joints and muscles. Individuals with FVIII deficiencies are typically treated by replacement or bypass therapies. Replacement therapies involve injection of FVIII concentrates from plasma or recombinant FVIII (rFVIII) expressed in mammalian cell lines. Bypass therapies, which are used in cases where an individual has developed inhibitors against FVIII, include activated prothrombin complex concentrates (aPCC) and recombinant factor VIIa (rFVIIa). These drugs “bypass” the generation of factor Xa (FXa) via the FVIIIa∶FIXa complex by promoting thrombin formation through elevating the plasma concentration of prothrombin and FXa (aPCC) or rFVIIa. Venous thrombosis appears to be rare in individuals with FVIII deficiencies and typically only occurs in association with indwelling venous catheters. In a review of all reported cases of non-catheter induced venous thrombosis in hemophilia, the most cited risk for thrombosis is treatment with bypassing agents [1]. The precise mechanisms of venous thrombosis has yet to be determined, however recent evidence suggests that tissue factor (TF) derived from endothelial adhered blood cells and microparticles likely plays a central role [2].

Previous in vitro flow assays studies with human blood show that severe FVIII deficiencies (<1% normal levels) or inhibition of FVIII results in smaller thrombi and reduced fibrin formation on collagen substrates at venous shear rates, but not arterial shear rates [3], [4]. Flow assays on animal derived subendothelium that contain TF also show a reduction in thrombus size and fibrin deposition for severe FVIII deficiencies at a shear rate of 650 s^−1^ [5]. Similarly, FVIII null mice have inhibited thrombi formation on collagen-TF substrates at venous shear rates, but not at arterial shear rates [6]. In the ferric chloride model, FVIII null mice formed unstable thrombi that could not occlude venules [7]. In the laser injury model, FVIII null mice formed smaller and less stable thrombi than wild type controls in venules [8]. These studies indicate that FVIII plays an important role in thrombus growth at venous shear stresses. However, these studies have only considered the absence of FVIII or FVIII levels of <1%, and therefore it is unknown how mild (1–5%) and moderate (5–30%) FVIII deficiencies affect thrombus formation under flow.

In this study, whole blood from a cohort of individuals with a wide range of FVIII levels (<1% to 26%) was perfused in microfluidic flow assays over collagen-TF substrates at venous flow conditions (100 s^−1^). We also measured the effect of treatment with recombinant FVIII (rFVIII) and recombinant factor VIIa (rFVIIa) on thrombus formation. In order to aid in the analysis of the experimental data, we used a spatial-temporal model of thrombus formation on immobilized TF to calculate thrombin generation [9]. Our results suggest that FVIII levels of >5% can support fibrin formation and that treatment with rFVIIa could potentially lead to a prothrombotic state.

## Methods

### Materials

L-α-phosphatidylcholine (PC) and L-α-phosphatidylserine (PS) were purchased from Avanti Polar Lipids (Alabaster, AL, USA). Texas red 1,2-dihexadecanoyl-sn-glycero-3-phosphoethanolamine (DHPE) was purchased from Invitrogen (Carlsbad, CA, USA). Lipidated and non-lipidated recombinant human tissue factor, recombinant double-chain tissue plasminogen activator, and an IMUBIND tissue factor ELISA Test Kit were purchased from American Diagnostica. Bio-Beads SM-2 were purchased from BioRad Laboratories (Hercules, CA, USA). Sodium deoxycholate was purchased from CalBiochem (Gibbstown, NJ, USA). Fibrillar collagen type 1 from equine tendon was from Chronolog Corp (Havertown, PA, USA). Alexa Fluor 488 protein labeling kit (Invitrogen, Carlsbad, CA, USA) was used to label fibrinogen according to the manufacturer's instruction. Pacific Blue anti-human CD41 (Biolegend, San Diego, CA, USA) was used to label platelets. Normal pooled plasma and FVIII deficient plasma was purchased from George King Bio-medical (Overland Park, KS). 16 well FAST slide incubation chambers were purchased from Whatman Inc. (Piscataway, NJ, USA) and used to pattern collagen and tissue factor on glass slides. Polydimethylsiloxane (PDMS) was used to fabricate microfluidic devices (Sylgard 184, Dow Corning, USA). HEPES buffered saline (HBS, 20 mM HEPES, 150 mM NaCl, pH 7.4) was made in house. All other reagents were purchased from Sigma-Aldrich (St. Louis, MO, USA).

### Preparation of lipidated tissue factor

TF was incorporated into liposomes using the method described by Smith and Morrissey [10]. Briefly, PC, PS and DHPE lipids stored in chloroform were dried under vacuum for one hour at an 80∶19∶1 molar ratio. The dried lipid film was then resuspended in 1 mL of 20 mM sodium deoxycholate in HBS, and allowed to hydrate for one hour at room temperature. Recombinant tissue factor (TF) was then added to the lipid mixture and incubated for 10 min. (8700∶1 lipid∶TF). Next, 50 mg of Bio-Beads slurry was added to the TF mixture to remove the sodium deoxycholate and agitated for 90 min. An additional 350 mg of Bio-Beads slurry was then added to the same mixture and agitated for another 90 min. Finally, the beads were allowed to settle, and the supernatant of liposomal TF was collected. The concentration of the lipidated TF was 461 nM as determined by ELISA.

### Patterning of prothrombotic substrates

Clean glass slides were inserted into incubation chambers with 16 wells with dimensions of 7 mm×7 mm×4 mm (L×W×D). Three adjacent wells were used to pattern patches of collagen and TF. First, 100 µL of 100 µg/mL type 1 fibrillar collagen was incubated in a well for one hour at room temperature. Following incubation, the collagen solution was removed and replaced with 100 µL of lipidated TF and allowed to incubate for 30 min. at room temperature. The stock lipidated TF (461 nM) was diluted to give surface concentrations of 0.23, 2.3 and 23 fmol TF/cm^2^. While the slides were still in the incubation chambers, they were rinsed three times with HEPES buffered saline (HBS). Finally, the entire slide was blocked in 5 mg/mL bovine serum albumin for one hour.

### Subject recruitment and blood collection

Subjects were recruited at the Hemophilia and Thrombosis Center of the University of Colorado Denver. The study and consent process received Institutional Review Board (IRB) approval from the University of Colorado Anschutz Medical Campus, and written informed consent was obtained for all participants. For participants under the age of 18, written informed consent was obtained from a parent or guardian. Participants consented to have information such as age, race and ethnicity shared in publications of research results. Phlebotomy was conducted in accordance with the Declaration of Helsinki and under the Colorado Multiple IRB. The treatment of patients with replacement and bypassing therapies was clinically indicated and independent of this study. Drs. Wang and Di Paola were responsible for providing treatment. Whole blood was collected via venipuncture into 3.2% sodium citrate.

### Laboratory phenotype of hemophilia A

Plasma FVIII activity levels were measured with a standard one-stage clotting assay (FVIII∶C) using a ST4 coagulometer (Diagnostica Stago). Patient whole blood was centrifuged for 15 minutes at 4°C and 2500× g, and the plasma supernatant was then centrifuged for an additional 15 minutes at the same settings to remove residual platelets. Normal pooled plasma (NPP) was obtained from a commercial source (George King Bio-Medical, Overland Park, KS). Samples were categorized by the percent of FVIII compared to NPP: severe (<1% FVIII), moderate (1–5%), mild (5–30%), and control (>50%).

### Replacement therapy with rFVIII

Four patients (referred to as patients 1–4) with severe hemophilia were treated with rFVIII and their pre and post (30 minutes after infusion) samples were drawn and evaluated in the microfluidic flow assay. Patient 1 is a 13 year old Caucasian male who received 41 IU/kg of rFVIII (Kogenate, Bayer). Patient 2 is a 19 year old Hispanic male who received 50 IU/kg of rFVIII (Helixate, CSL Behring). Patient 3 is a 13 year old Caucasian male who received 27 IU/kg of rFVIII (Kogenate, Bayer). Patient 4 is a 4 year old Caucasian male who received 24 IU/kg or rFVIII (Advate, Baxter).

### Bypass therapy with rFVIIa

Two patients (referred to as patients 5 and 6) with severe hemophilia and history of high inhibitors were treated with rFVIIa and their pre and post (30 minutes after infusion) samples were drawn and evaluated in the microfluidic flow assay. Patient 5 is a Caucasian male with an inhibitor titer of 7.2 Bethesda Unites (BU). Patient 6 is a Hispanic male who had a titer of 3.1 BU. Both patients received a dose of 90 µg/kg of rFVIIa (NovoSeven, Novo Nordisk).

### Microfluidic flow assays

The design and operation of the microfluidic flow assay followed previous protocols with a few minor changes [11]. A device consisting of three channels with a height of 100 µm and a width of 500 µm was reversibly vacuum bonded to a patterned collagen-TF substrate on a glass slide. The flow rate was set in a syringe pump to achieve the desired wall shear rate. Blood was incubated for 10 min. with a non-function blocking Pacific Blue antihuman CD41 antibody. Exogenous AlexaFluor488 labeled human fibrinogen was added to the whole blood at a molar ratio of 100∶1 (plasma∶labeled) assuming a fibrinogen plasma concentration of 3 mg/mL. Whole blood was recalcified with 7.5 mM CaCl_2_ immediately before the assays. Whole blood or plasma (normal pooled or FVIII deficient) was perfused through the channels for 5 min. at a wall shear rate of 100 s^−1^ or 1000 s^−1^. The accumulation of platelets and fibrin was monitored at the upstream edge of the collagen-TF patch using an inverted fluorescence microscope (40X, NA 0.6, Olympus IX81) equipped with a 16-bit CCD camera (Orca-R2, Hamamatsu). An image was captured in each channel every 10 sec. over the duration of the experiment. After whole blood perfusion, a wash buffer (HBS, 2 mM CaCl_2_, 1 U/mL heparin) was perfused through the channel for 5 min. at a shear rate of 100 s^−1^ and then samples were prepared for D-Dimer measurements or scanning electron microscopy.

### D-dimer assay

A plasmin solution (288 µg/ml in HBS) was perfused through the microfluidic channel at a flow rate of 5 µL/min for 10 minutes, and then flow was stopped for 10 minutes to allow for any remaining fibrin to be digested. The plasmin solution was collected from the device and snap frozen and stored at −70°C. D-dimer levels were measured by ELISA (American Diagnostica) according to the manufacturers instructions at a dilution factor of 1∶10 (digested fibrin∶diluent).

### Image analysis

Platelet aggregate area and surface coverage was calculated using previously described custom image analysis routines [11]. For fibrin density, a disk structuring element decomposition was used to estimate the background fluorescence [12]. The background was subtracted from the raw image and the integrated fluorescence (sum of each pixel) of the entire image was normalized by the image area.

### Scanning electron microscopy

Samples were prepared as previously described [13]. Samples were imaged by scanning electron microscopy (JEOL 7000) at accelerating voltage of 1.5 kV and a working distance of 6 mm.

### Spatial-temporal model of thrombus formation

The two-dimensional spatial-temporal model consists of partial differential equations for the concentration of platelets and coagulation chemicals that evolve under flow [9]. The differential equations, physical properties and rate constants are given in Tables S1–S8 in Text S1). The rectangular domain in which the equations are solved represents a segment of blood vessel with 60 µm height, 240 µm length, and wall shear rate of 100 s^−1^. A 90 µm portion of the bottom wall is considered injured, exposing subendothelium-bound TF and collagen to the flowing blood. The exposed TF initiates coagulation reactions and the collagen allows platelets to adhere. Platelets activated by thrombin via coagulation also adhere at the injury site and a porous thrombus forms. The porous thrombus physically hinders the flow of plasma within the thrombus relative to the free stream velocity outside of the thrombus.

### Statistical analysis

Correlation coefficients were calculated using the Spearman statistic. Kruskal-Wallis ANOVA was used to determine differences (p<0.01) between clinical groups, followed by a post hoc Tukey's honestly significant difference test to determine differences between pairs. The Mann-Whitney U-test was used to determine differences (p<0.01) between fibrin dynamics metrics before and after replacement and bypassing treatments.

## Results

### Sensitivity of fibrin accumulation to surface TF concentration and shear rate

In order to model venous thrombosis, we determined the TF surface concentration that would induce measurable fibrin formation. Whole blood from normal donors was perfused at 100 s^−1^ over collagen-lipid surfaces with a TF surface concentration of 0, 0.23, 2.3 and 23 fmol TF/cm^2^ (Fig. S1). The total lipid concentration and collagen concentration (100 µg/mL) was held constant. There was no difference in fibrin accumulation between no TF and 0.23 fmol TF/cm^2^, demonstrating that this concentration was below the threshold level needed to induce fibrin formation in agreement with previous results [14]. At 2.3 fmol TF/cm^2^ there was a measureable amount of fibrin deposition. At 23 fmol TF/cm^2^ the amount of fibrin accumulation occluded the channel during most assays. Based on these data we used 2.3 fmol TF/cm^2^ for all experiments with hemophilia and healthy control samples. The relationship between fibrin density as measured by fluorescence intensity (FI) and D-dimer levels was linear on surfaces of 0–23 fmol TF/cm^2^: D-dimer (µg/mL) = 1.3×FI(RFU)−9.7, R^2^ = 0.95. Therefore, we assume that the linear relationship between fluorescence intensity and fibrin deposition on 2.3 fmol TF/cm^2^ holds for normal and FVIII deficient samples. Whole blood from normal donors perfused over 2.3 fmol TF/cm^2^ at 1000 s^−1^ resulted in no observable fibrin fibers by fluorescence or D-dimer levels significantly different from perfusion over collagen substrates in the absence of TF (data not shown). This surface concentration of TF is below the threshold concentration necessary to induce fibrin formation at 1000 s^−1^ [14], and consequently experiments with hemophilia samples were only conducted at 100 s^−1^.

### Fibrin morphology in thrombi with FVIII deficiencies

**Figure 1:**
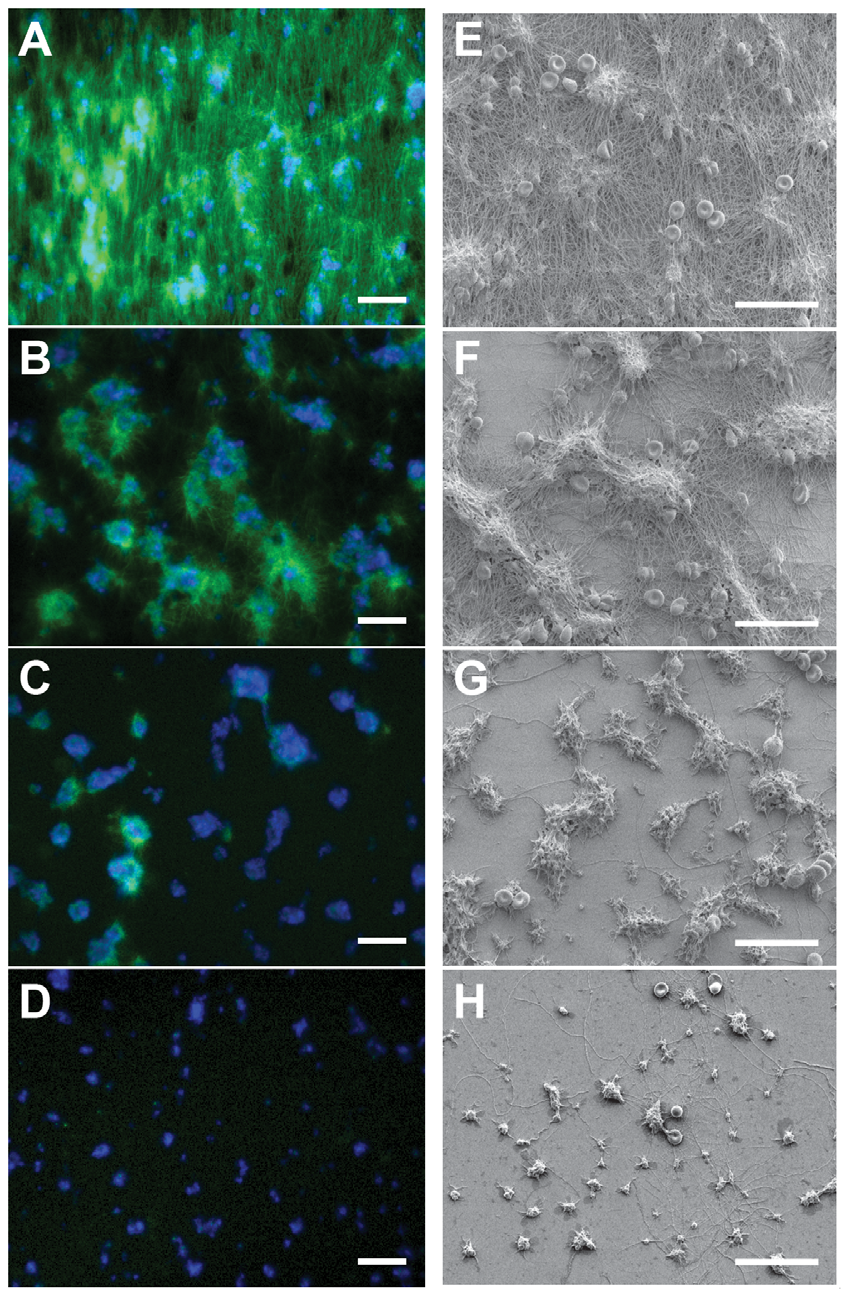
Thrombi formed under flow on collagen-TF surfaces from individuals with FVIII deficiencies. Recalcified whole blood was perfused over glass slides coated with 2.3/cm^2^ and type 1 fibrillar collagen at 100 s^−1^ for 5 min. Representative images of platelets (*blue*, anti-CD41) and fibrin(ogen) (*green*, Alexa488-fibrinogen) accumulation for a normal control (A) and hemophilia samples with plasma FVIII levels of 11.1% (B), 3.1% (C), and 0.4% (D) at 5 min. Scale bar = 25 µm. Scanning electron micrographs of thrombi from the same individuals; (E) 100% 11.1% (F), 3.1% (G), and 0.4% (H). Platelet aggregates are immersed in a fibrin mesh for the control (E) and form a starburst like pattern for mild hemophilia samples (F). The fibers on the surface in (G) and (H) are collagen fibers. Scale bar = 25 µm.

Whole blood samples from 20 HA patients (FVIII∶C range 0.4–26.1%) and 9 healthy controls were used for this study (Table 1). For each sample, platelet and fibrin accumulation was monitored over the course of 5 min. at a wall shear rate of 100 s^−1^. Accumulation of fibrin and platelets is shown in Fig. 1 for a control subject and individuals with mild, moderate and severe hemophilia.

**Table 1:** Clinical characteristics of hemophilia A patients and controls.

Category†	FVIII∶C (% of NPP)	Fibrin density (RFU) avg (stdev)	Platelet aggregate size (µm^2^) avg (stdev)
Control	320.3	33 (11)	141 (23)
Control	261.7	36 (3)	90 (20)
Control	247.9	43 (5)	115 (35)
Control	229.0	38 (5)	129 (33)
Control	215.0	36 (7)	121 (44)
Control	191.1	39 (10)	80 (14)
Control	138.4	41 (6)	131 (38)
Control	122.8	38 (8)	75 (20)
Control	84	40 (4)	86 (21)
Mild	26.1	36 (3)	111 (11)
Mild	22.4	27 (2)	98 (20)
Mild	18	27 (1)	88 (13)
Mild	11.1	23 (4)	119 (34)
Mild	8.3	24 (4)	165 (41)
Moderate	4.6	15 (5)	60 (10)
Moderate	4.3	13 (4)	66 (14)
Moderate	3.1	12 (3)	55 (9)
Moderate	3.1	22 (3)	40 (10)
Moderate	2.7	12 (4)	72 (22)
Moderate	2.4	5 (3)	50 (15)
Moderate	2.3	6 (2)	61 (20)
Moderate	1.9	10 (3)	81 (18)
Severe	1.0	8 (3)	26 (6)
Severe	0.9	9 (4)	29 (9)
Severe	0.8	10 (2)	17 (5)
Severe	0.5	8 (3)	15 (6)
Severe	0.5	11 (3)	27 (8)
Severe	0.4	11 (2)	16 (3)
Severe	0.3	12 (3)	29 (4)

FVIII∶C was measured by a one-stage clotting assay and expressed as percent of normal pooled plasma (NPP). Fibrin density and platelet aggregate size were measured at the end of a 5 min. flow assay on type I collagen and 2.3 fmol TF/cm^2^ at 100 s^−1^. Data is presented as the average and standard deviation of n = 3.

† Categories are based on FVIII∶C where control >50%, mild 5–30%, moderate 1–5%, and severe <1%.

**Figure 2:**
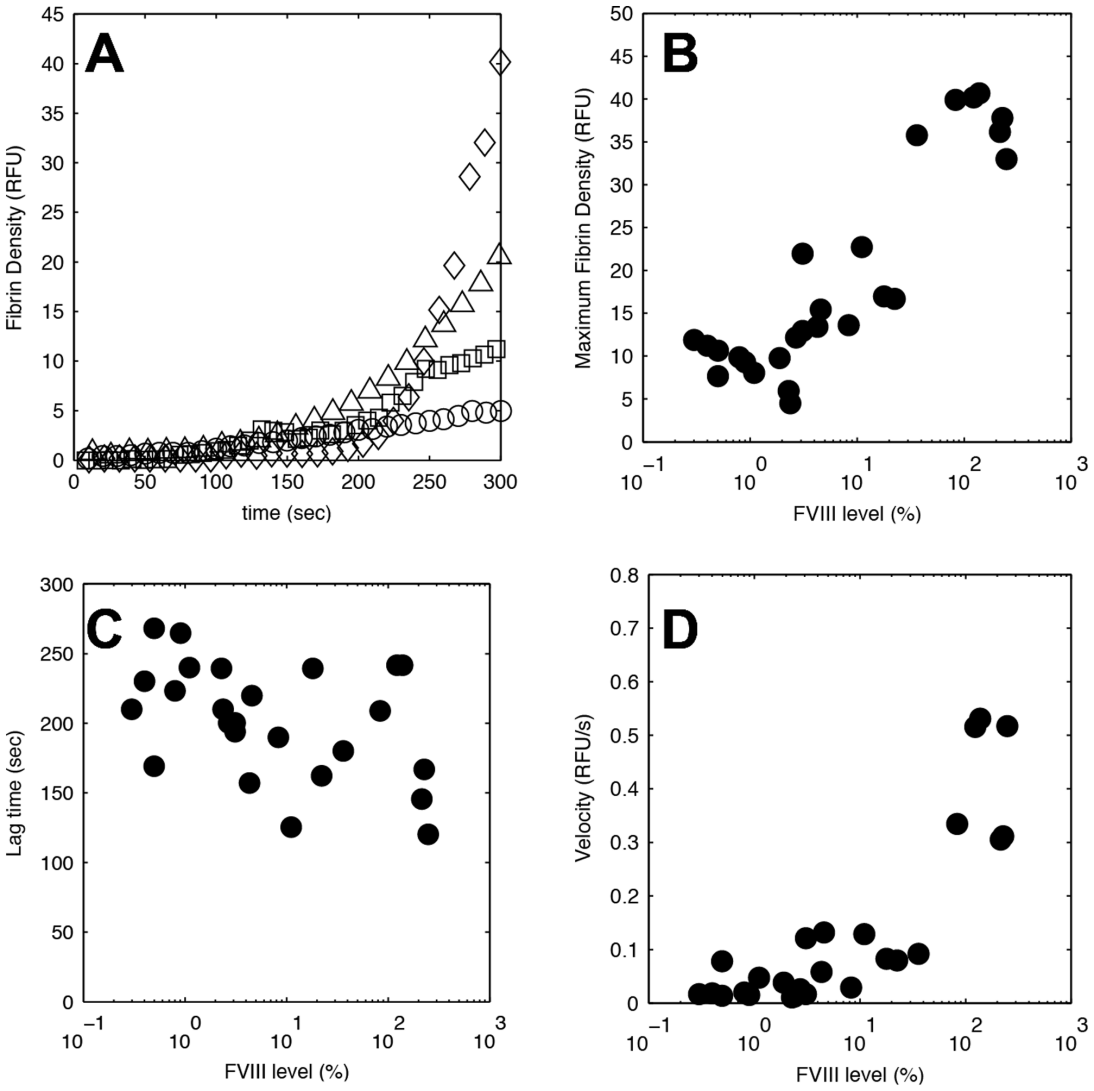
Fibrin deposition dynamics from individuals with FVIII deficiencies. (A) Fibrin density as a function of time for a normal control (◊) and hemophilia samples with plasma FVIII levels of 11.1% (▵), 3.1% (□), and 0.4% (○). The dynamics of fibrin deposition were quantified by three metrics: (B) Maximum fibrin density, which is the integrated fluorescence of the fibrino(gen) signal at the end of the 5 min. assay. (C) The lag time, which is the time to 10% of the maximum fibrin density for normal controls (4 RFU). (D) The velocity, which is the slope of the fibrin density curve from the lag time to the end of the assay. Each data point (•) represents a single individual with either normal or deficient FVIII levels.

In control subjects, fibrin slowly accumulated on and around platelet aggregates in the first two to three minutes and then spread to the entire field of view by 5 min. (Fig. 1A, Video S1). At approximately 3 min., there was a secondary burst of fibrin accumulation that was observed in all control subjects (Fig. 2). Fibrin formation started in a starburst pattern, and at later times (>3 min.), fibers tended to align with the direction of flow. Electron microscopy revealed a network of fibers that was densest near platelet aggregates but that covered the entire surface (Fig. 1E). Only in control subjects were fibrin networks observed outside of the areas adjacent to platelet aggregates.

Mild FVIII deficiencies (5–30% FVIII) had similar fibrin density and accumulation rates at early times (<3 min.) as control subjects (Fig. 2). However, there was no secondary burst of fibrin at later times (>3 min.), just a steady accumulation adjacent to platelet aggregates (Video S2). The fibrin in mild samples was most dense on and near the periphery of platelet aggregates (Fig. 1B). Electron micrographs of mild hemophilia samples show a starburst pattern of fibrin fibers emanating from platelet aggregates (Fig. 1F).

In moderate FVIII deficiencies (1–5% FVIII), little to no fibrin was observed around platelet aggregates (Fig. 1C). There was a slight increase in the accumulation rate in the first two minutes, after which there was no evidence of further fibrin formation (Fig. 2, Video S3). Electron micrographs confirm that there are few, if any, large (>50 nm) fibrin fibers on or near platelet aggregates (Fig. 1G).

Similar to moderate deficiencies, in severe FVIII deficiencies (<1%) there were little to no fibrin fibers observed (Fig. 1D, Video S4). The accumulation of fibrin(ogen) was modest and generally did not significantly change after the first three minutes of the assay (Fig. 2A). The overlay between the labeled platelets and the labeled fibrin(ogen) was almost identical in every case (Fig. 1D), suggesting that the observed signal is likely platelet bound fibrinogen. There was no evidence of fibrin fibers on or near platelet aggregates by electron microscopy (Fig. 1H).

Platelet adhesion preceded fibrin formation for all normal and patient samples. To test whether platelets are necessary for fibrin fiber formation we ran a set of experiments with normal pooled plasma (NPP) and FVIII deficient platelet poor plasma at 100 s^−1^. We observed no fibrin fibers or accumulation of fluorescence signal above background in either case in the middle of the channel over 5 min. In NPP we did observe fibers in the corners of channels, which is a result of accumulation of coagulation products in the low flow areas near the corners of rectangular channels. No fibers were observed anywhere in the channel with FVIII deficient plasma.

### Fibrin deposition dynamics as a function of FVIII levels

We used three metrics to quantify the dynamics of fibrin formation; (i) the maximum fibrin density, (ii) the lag time to 10% of maximum fibrin formation for normal subjects (38±4 RFU), and (iii) the velocity of fibrin accumulation defined as the slope of the line defined by the lag time and the time to maximum fibrin density (Fig. 2). Both the maximum fibrin density (Fig. 2B, r = 0.80, p<10^−6^) and deposition velocity (Fig. 2D, r = 0.85, p<10^−7^) were strongly correlated to FVIII levels. The lag time was inversely correlated to FVIII levels (Fig. 2C, r = −0.41, p = 0.04), although the dependence was not statistically significant.

### Clinical phenotype and platelet and fibrin accumulation

**Figure 3:**
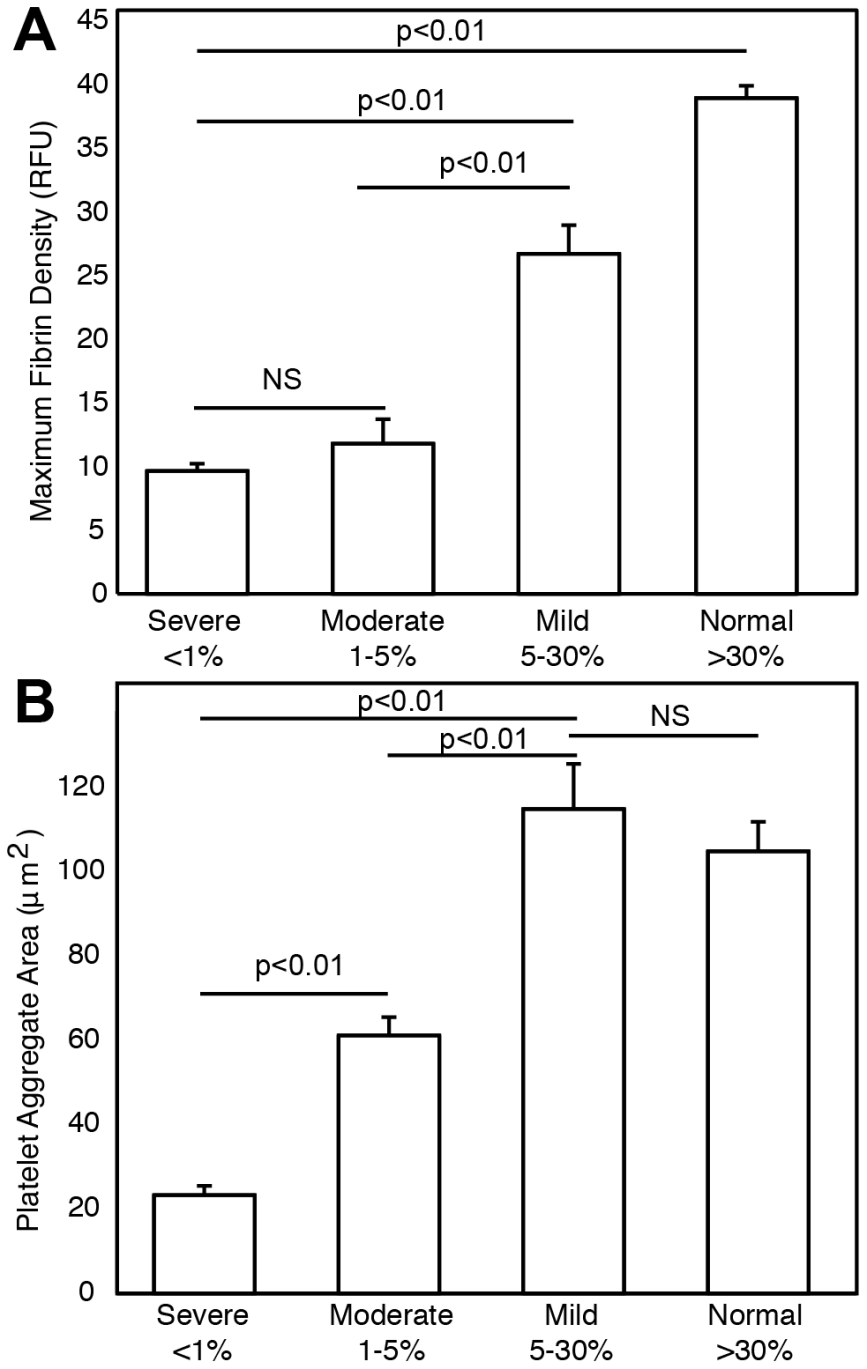
Fibrin density and platelet aggregate size for different clinical categories of hemophilia A. (A) Maximum fibrin density and (B) platelet aggregate area for severe, moderate and mild FVIII deficiencies compared to normal controls. Error bars represent standard error. Lines indicate comparisons between pairs according to Tukey's honestly significantly difference test following Kruskal-Wallis ANOVA. NS indicates not significant.

Fibrin accumulation was supported in mild HA, but it was significantly less than for normal FVIII levels (Fig. 3A). There was not a significant difference in maximum fibrin density between severe and moderate HA. However, platelet aggregates were significantly larger in moderate HA than severe HA (Fig. 3B). This result suggests that the thrombin concentration was high enough to activate platelets in moderate HA, but not to support fibrin formation.

### Thrombin generation dynamics as a function of FVIII levels

**Figure 4:**
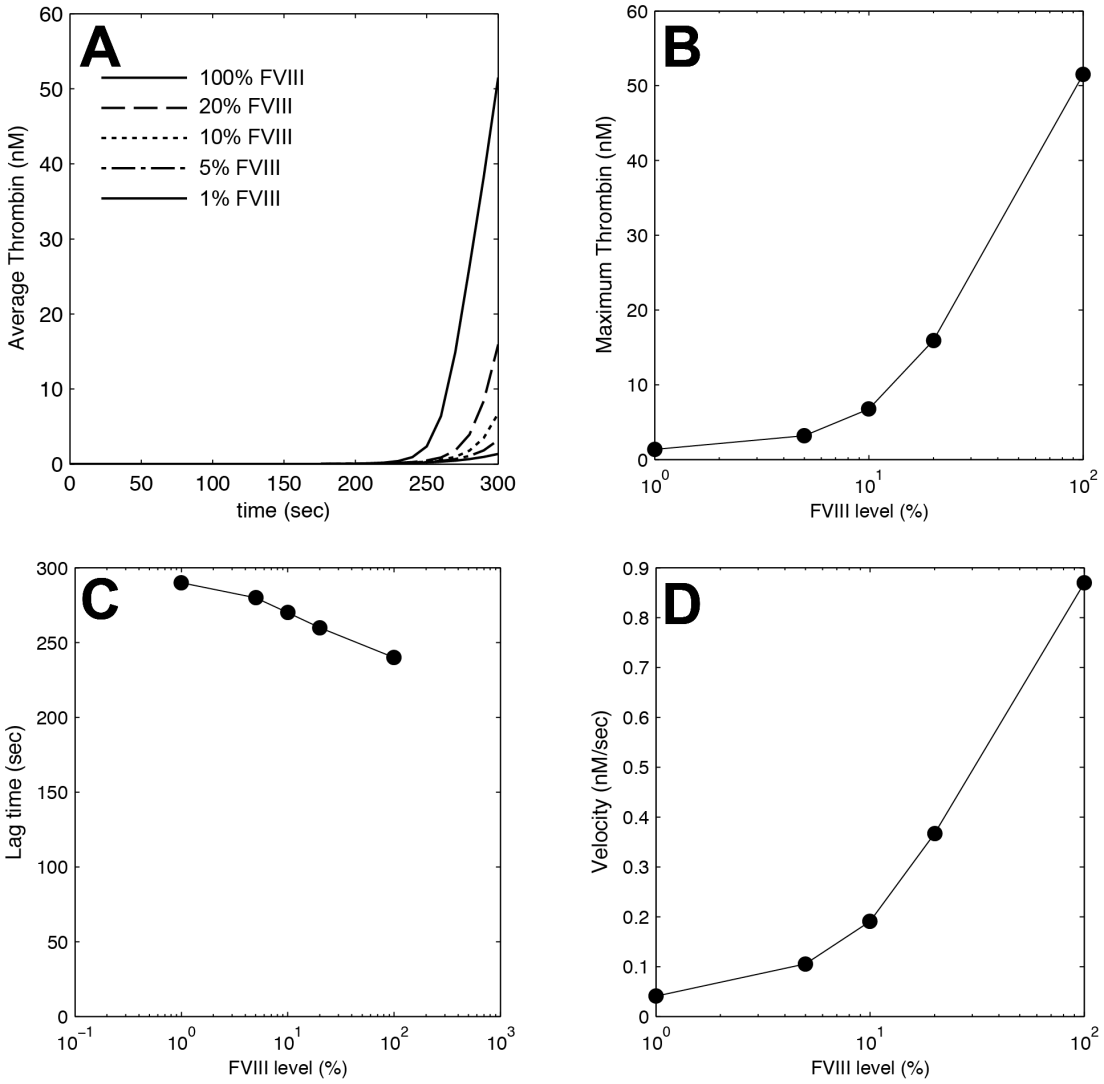
Thrombin generation under flow in FVIII deficiencies. The average thrombin concentration within a thrombus was calculated using a spatial-temporal computational model of thrombus formation on 2.3 fmol TF/cm^2^. (A) Average thrombin concentration as a function of time for FVIII levels of 1, 5, 10, 20, and 100%. The dynamics of thrombin generation were quantified by three metrics: (B) Maximum thrombin concentration, which is the thrombin concentration at the end of the 5 min. simulation. (C) The lag time, which is the time to 1 nM thrombin. (D) The velocity, which is the slope of the average thrombin curve from the lag time to the end of the simulation. Each data point (•) represents a single simulation. The lines are extrapolations between simulation data points.

A computational model of thrombus formation was used to estimate the effect of FVIII on thrombin generation. Thrombin generation was characterized by the average thrombin concentration (Fig. 4) within the thrombus and by the cumulative thrombin produced, which includes both the thrombin within the thrombus and the thrombin washed away by the flow. The trends are similar for both, so we focus on the average thrombin generation here, but the data for cumulative thrombin production can be found in Fig. S2. We used similar metrics to quantify the dynamics of thrombin formation as fibrin formation; (i) the maximum thrombin concentration, (ii) the lag time to 1 nM thrombin (concentration that activates platelets through PAR1 [15]), and (iii) the velocity defined as the slope of the line defined by the lag time and the time to maximum thrombin concentration.

The calculated thrombin generation closely matches the trends in experimentally measured fibrin deposition. Platelets begin to adhere at 200–250 sec, followed by a burst in thrombin generation that diminishes with decreasing FVIII levels. Maximum thrombin concentration ranges from 50 nM at 100% FVIII to 1 nM at 1% FVIII. There was approximately a 5-fold decrease in both thrombin velocity and fibrin velocity between 100% and 10% FVIII. The velocity of both thrombin and fibrin continues to decrease between 10% and 1% FVIII, but at a more modest rate. Thus, the model would predict that FVIII levels from 5–10% could possibly present more like a mild phenotype than a moderate phenotype. However, since we only have one patient in that range it is difficult to draw any conclusions based on the flow assay experiments. The lag time for fibrin and thrombin dynamics agree qualitatively; there is modest decrease in the lag time with increasing FVIII levels.

**Figure 5:**
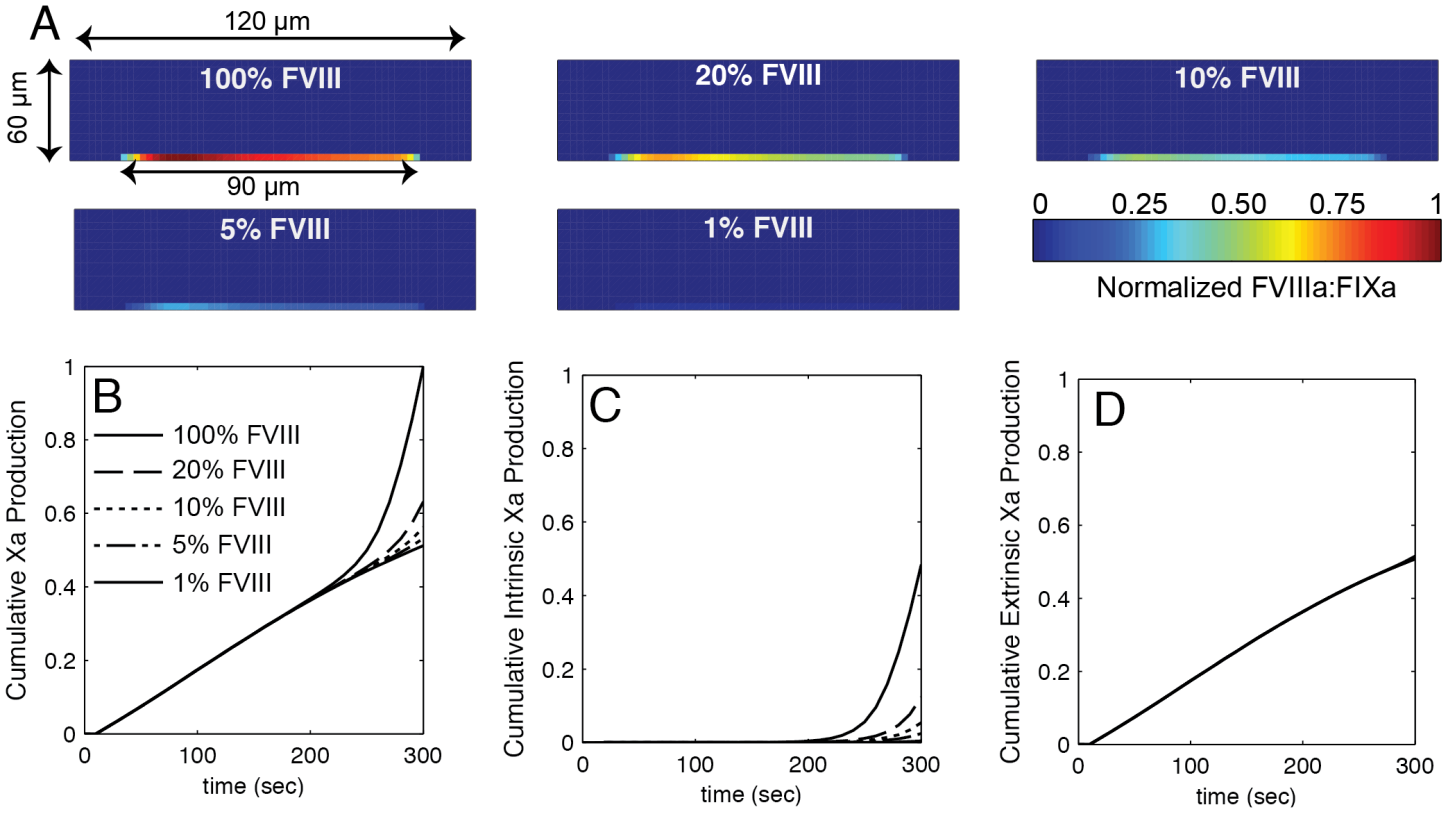
The relative roles of intrinsic and extrinsic tenase on Xa generation. (A) The distribution of intrinsic tenase (FVIIIa∶FIXa) within the thrombus for different FVIII levels. The concentration is normalized by the maximum intrinsic tenase concentration in a thrombus formed at 100% FVIII. The thrombus forms on 90 µm patch of TF on the bottom wall. (A) The total cumulative Xa production for different FVIII levels (B) and the relative contribution from intrinsic tenase (C) and extrinsic (TF∶FVIIa) tenase (D). Xa production is normalized by the total cumulative production of Xa for 100% FVIII.

Reduced thrombin generation for FVIII deficiencies can be traced to reduction in intrinsic tenase (FVIIIa∶FIXa). Fig. 5A shows the spatial distribution of intrinsic tenase as a function of FVIII levels. Fig. 5B–D shows the total, platelet-derived and wall-derived FXa production. Wall derived FXa is produced solely by extrinsic tenase (TF∶VIIa). Platelet derived FXa is produced solely by intrinsic tenase. Prior to substantial platelet adhesion (<200 sec), all FXa is produced on the wall through extrinsic tenase in a FVIII independent manner. Following platelet adhesion (200–300 sec), FXa production rapidly increases through intrinsic tenase. In all cases of FVIII deficiency, extrinsic tenase is the major source of FXa.

### Fibrin and thrombin generation in response to rFVIII and rFVIIa therapies

**Figure 6:**
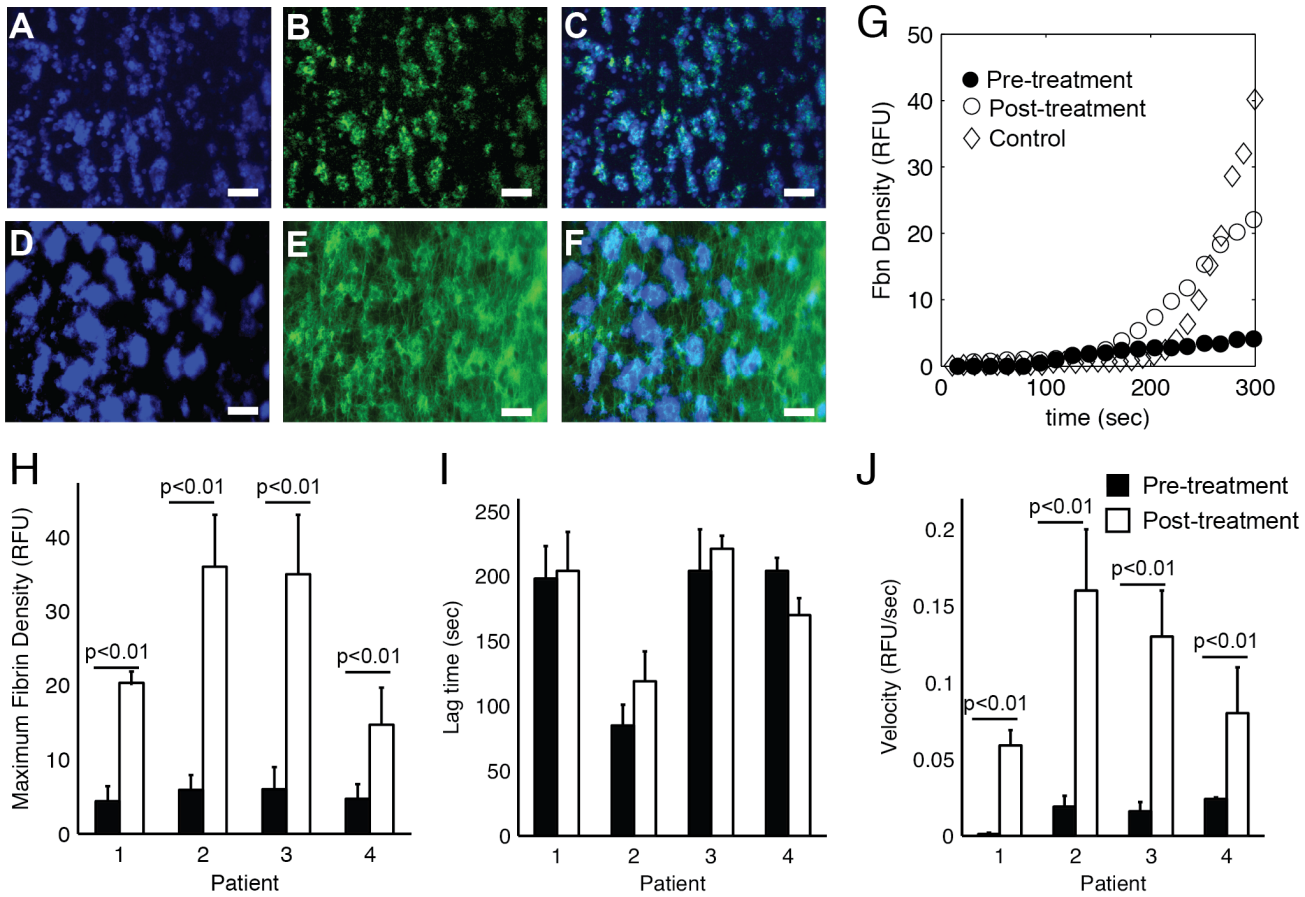
Fibrin deposition dynamics in response to rFVIII treatment. Four patients (1–4) with severe hemophilia where treated with rFVIII (see Methods for doses). Recalcified whole blood was perfused over glass slides coated with 2.3 fmol TF/cm^2^ and type 1 fibrillar collagen at 100 s^−1^ for 5 min before and 30 min after treatment with rFVIII. Platelets (*blue*, anti-CD41), fibrin(ogen) (*green*, Alexa488-fibrinogen) and their overlay immediately before (A–C) and 30 min. after (D–F) rFVIII injection. (G) Transient fibrin density pre-treatment (•) and post-treatment (○) in comparison to a normal control (◊). The dynamics of fibrin deposition before (black bars) and after (white bars) was characterized by (H) maximum fibrin density, (I) the lag time, and (J) the velocity. Error bars represent standard deviations of n = 3. Lines indicate comparisons between pairs according to the Mann-Whitney U-test.

Platelet and fibrin accumulation was measured from four individuals with severe FVIII deficiency on prophylactic replacement therapy before and 30 min. after injection of rFVIII. Representative images of final platelet and fibrin accumulation before and after treatment are shown in Fig. 6A–F. The dynamics of fibrin accumulation for patient 1 is shown in Fig. 6G and shows that treatment with rFVIII normalizes fibrin formation. In all cases there was a significant increase in maximum fibrin density and velocity after treatment (Fig. 6H, 6J). There was no difference in lag time before and after treatment (Fig. 6I). Patients 2 and 3 had post-treatment FVIII activity levels of 101% and 79%, respectively, and showed fibrin accumulation in the flow assays that was similar to control samples. Patients 1 and 4 had post-treatment FVIII activity levels of 46% and 38%, respectively, and fibrin accumulation was slightly less than what was observed in healthy control samples (38±4 RFU).

**Figure 7:**
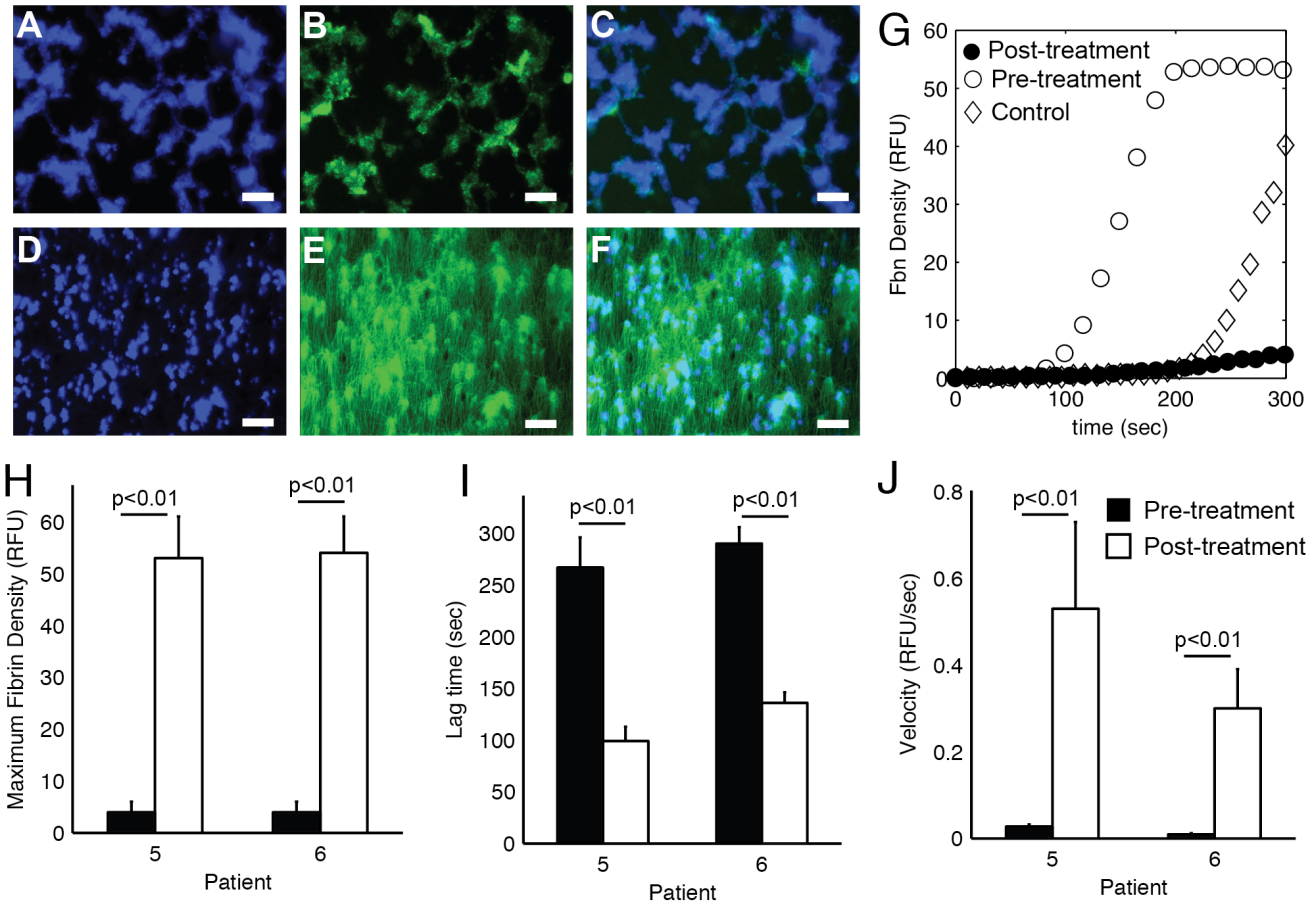
Fibrin deposition dynamics in response to rFVIIa treatment. Two patients (5–6) with severe FVIII deficiency with high inhibitor titer were treated with 90 µg/mL rFVIIa. Recalcified whole blood was perfused over glass slides coated with 2.3 fmol TF/cm^2^ and type 1 fibrillar collagen at 100 s^−1^ for 5 min before and 30 min after treatment with rFVIIa. Platelets (*blue*, anti-CD41), fibrin(ogen) (*green*, Alexa488-fibrinogen) and their overlay immediately before (A–C) and 30 min. after (D–F) rFVIIa injection. (G) Transient fibrin density pre-treatment (•) and post-treatment (○) in comparison to a normal control (◊). The dynamics of fibrin deposition before (black bars) and after (white bars) was characterized by (H) maximum fibrin density, (I) the lag time, and (J) the velocity. Error bars represent standard deviations of n = 3. Lines indicate comparisons between pairs according to the Mann-Whitney U-test.

Platelet and fibrin accumulation was measured in two individuals with severe FVIII deficiency with inhibitors before and 30 min. after treatment with rFVIIa. Representative images of final platelet and fibrin accumulation before and after treatment are shown in Fig. 7A–F. Treatment with rFVIIa changed the dynamics of fibrin formation in two ways (Fig. 7G). First, there was a dramatic decrease in lag time from 267 sec to 99 sec in patient 5 and 290 sec to 136 sec in patient 6 (Fig. 7I). Second, the maximum fibrin density following rFVIIa treatment was 39% and 42% higher than the average maximum fibrin density from healthy controls (Fig. 7H). The velocity of fibrin accumulation was within the range observed for healthy controls (Fig. 7J).

**Figure 8:**
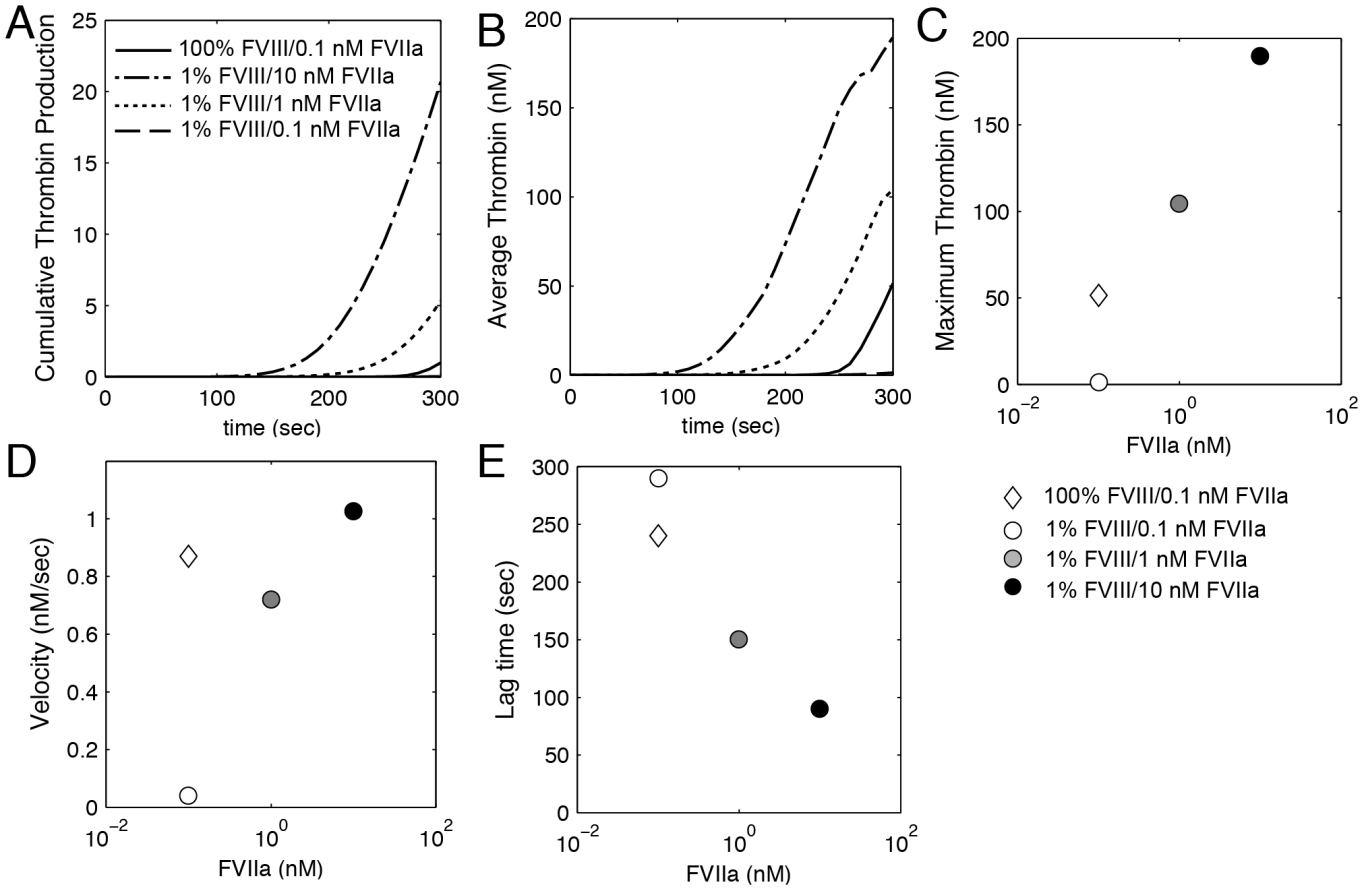
Thrombin generation under flow for rFVIIa treatment. The computationally calculated cumulative thrombin production (A), average thrombin concentration (B), maximum thrombin concentration (C), velocity of thrombin production (D) and lag time (E) for 1% FVIII levels at 0.1, 1, and 10 nM FVIIa plasma concentration compared to 100% FVIII levels and 0.1 nM FVIIa. The cumulative thrombin production is normalized by the maximum for 100% FVIII, 0.1 nM FVIIa.

We simulated the effect of 0.1, 1 and 10 nM FVIIa in the plasma on thrombin generation for 1% FVIII levels (Fig. 8). In agreement with experimentally measured fibrin formation, the addition of FVIIa decreases lag time and increases thrombin production and average thrombin concentration. The thrombin lag time decreases from 290 sec at the endogenous FVIIa level (0.1 nM) to 150 sec and 90 sec for 1 nM and 10 nM (range of anticipated plasma concentration following 90 µg/mL dose), respectively. The average thrombin concentration exceeds that of normal levels (100% FVIII, 0.1 nM FVIIa) by 202% and 368% for 1 nM and 10 nM, respectively. This enhancement in thrombin generation is almost completely driven by extrinsic tenase (Fig. S3). There is roughly a 2-fold and 4-fold increase in Xa production for 1 nM and 10 nM FVIIa, respectively.

## Discussion

In this study we used microfluidic and computational models to measure thrombus formation on a well-defined collagen-TF surface under venous conditions in cohort of patients representing all three clinical phenotypes of HA (severe, moderate, mild). We also measured the response to replacement and bypassing therapies in individuals with severe FVIII deficiencies. This combined experimental and computational approach yields new insights into the biophysical mechanisms that regulate thrombus formation in HA. In comparison to static conditions, flow can either limit or enhance local enzyme concentrations and fibrin polymerization reactants [13], [16], [17]. In the case of FVIII deficiencies, flow dilutes thrombin such that reduction in thrombin generation is more severe than under static conditions. For example, there is a 62% reduction of peak thrombin concentration between 100% and 1% FVIII levels under static conditions [18] compared to a 98% reduction under flow observed in this study. Similarly, a 69% reduction in the rate of fibrinopeptide A release in severe HA compared to normal controls was reported in a static whole blood coagulation assay [19], compared to an average 94% reduction in fibrin deposition velocity under flow. On the other hand, convective transport by flow increases the flux of rFVIIa to the TF-rich surface, and thus decreases the lag time to thrombin generation and fibrin formation much faster than by diffusion alone.

Fibrin formation was supported by mild FVIII deficiencies, but not moderate or severe deficiencies. This is a novel observation that supports clinical evidence that individuals with mild FVIII deficiency (5–40%) do not experience bleeding except after severe trauma or surgery and that venous thrombosis is rare in these individuals [20]. Computations predict a local thrombin concentration of 5–20 nM over FVII levels of 5–40%. Based on previous studies, this thrombin concentration can support fibrin formation under flow, but only at wall shear rates of 10–25 s^−1^ [13]. Under static conditions, fibrin formation is supported at thrombin concentration of less than 1 nM. The shear rate may be lower in and near the nascent thrombus due to perturbations in the flow field by platelets that protect coagulation products from dilution by flow. This reasoning is supported by the observation that platelet adhesion preceded fibrin formation under all conditions. Even a very high surface TF concentration (23 fmol TF/cm^2^) could not support fibrin formation in the absence of platelets at 100 s^−1^. Thrombus formation on homogenized atherosclerotic plaques containing both collagen and TF demonstrate similar trends; platelet accumulation precedes fibrin formation and no fibrin is observed in the absence of platelets [21]. Platelet aggregate size was larger in moderate FVIII deficiencies compared to severe deficiencies. This observation suggests that while the local thrombin concentration in moderate deficiencies was unable to support fibrin deposition, it was high enough to activate platelets.

Treatment of FVIII deficiency with replacement therapy normalized fibrin deposition, while treatment with bypass therapy significantly altered fibrin deposition dynamics compared to healthy controls. For patients receiving replacement therapy (rFVIII), the post-treatment FVIII activity was equal to or less than normal FVIII activity. Therefore, the observation that fibrin deposition was equal to or slightly less than the normal control is expected. For the two patients receiving bypass therapy (rFVIIa), a decreased lag time and increased cumulative fibrin deposition were observed. The computational model predicts faster assembly of the TF∶FVIIa complex owing to a higher flux of FVIIa being delivered to the surface compared to endogenous FVIIa levels. In the absence of TF, previous studies have shown that adding 13 nM rFVIIa enhances the final accumulation of platelets on collagen at 1600 s^−1^, but does not affect the lag time [22]. The increase in platelet accumulation is thought to be a function of rFVIIa binding to platelets followed by subsequent activation of FX [23]. In our computational model, we did not account for rFVIIa binding to platelets and still found that 1–10 nM of FVIIa could boost thrombin generation significantly through extrinsic Xase. The contribution of platelet bound rFVIIa on fibrin formation under flow requires further study. Nevertheless, our experimental and computational results suggest that treatment of FVIII deficiency with rFVIIa could lead to prothrombotic risks in agreement with clinical observations [24].

There is growing appreciation for the utility of computational models in modeling coagulation and platelet function, in that they can integrate the details of complex phenomena to yield mechanistic insight or to test novel hypotheses [25]–[28]. Perhaps in the future these computational models will allow scientists and clinicians to be able to predict the degree of hemostatic responses in patients with clotting factor deficiencies. In this study we used a computational model of thrombus formation that incorporates platelet adhesion, aggregation and coagulation to predict the effect of perturbations to coagulation (FVIII deficiency and rFVIIa treatment) on thrombin generation. There was agreement between the computationally predicted thrombin and experimentally measured fibrin dynamics in terms of lag time, maximum concentration, and rate of production. The lag time was associated with the time for substantial platelet adhesion (>3–4 min) and was relatively insensitive to FVIII levels in comparison to FVIIa levels, which dramatically decreased lag times. The maximum thrombin concentration and fibrin density decreased five-fold between 100% and 10% FVIII levels. The fibrin density was less sensitive to FVIII levels below 10% than the thrombin concentration due to the background fluorescence caused by labeled fibrinogen. However, there was a decrease in platelet aggregate size between 1% and 10% levels that corresponds with the predicted drop in local thrombin concentrations that approach subthreshold concentrations of thrombin (∼1 nM) for activating PAR1 [15]. While the mechanisms that regulate thrombin generation and fibrin formation are different, observations from in vivo and in vitro assays using a platelet bound thrombin sensor show that thrombin and fibrin co-localize in the interior of a clot [29]. This suggests that fibrin is a good proxy for thrombin concentration in flow-based assays.

In summary, FVIII deficiencies can profoundly influence thrombin and fibrin formation on TF-rich substrates at venous shear rates. The primary effect of FVIII deficiency is that the rate of thrombin generation becomes slower on the platelet surface and thus the characteristic burst of thrombin is absent. Under flow, the situation is further exacerbated because the local thrombin concentration is diluted more quickly than by diffusion alone. In the case of mild FVIII deficiencies and in the presence of high surface TF concentrations, the local thrombin concentration is sufficient to promote fibrin formation in and adjacent to platelet aggregates. Platelets play a central role in both providing a burst in thrombin production and in providing a physical shelter for coagulation reaction and fibrin formation. Treatment of severe FVIII deficiency with rFVIIa gives thrombi that form faster and accumulate more fibrin than healthy controls.

## Supporting Information

Figure S1 **Sensitivity of fibrin deposition to tissue factor surface concentration.** (A) Fibrin density as measured by integrated fluorescence resulting from perfusions of normal whole blood at 100 s^−1^ for 5 min over surfaces with varying surface TF concentrations. (B) D-Dimer levels from plasmin digested thrombi formed at 100 s^−1^ for 5 min over surfaces with varying surface TF concentrations.
(EPS)

Figure S2 **Thrombin generation under flow in FVIII deficiencies.** The cumulative thrombin production was calculated using a spatial-temporal computational model of thrombus formation on 2.3 fmol TF/cm^2^. All data is normalized by the maximum cumulative thrombin production for 100% FVIII. (A) Normalized cumulative thrombin as a function of time for FVIII levels of 1, 5, 10, 20, and 100%. The dynamics of thrombin generation were quantified by three metrics: (B) Maximum cumulative thrombin production, which is the total thrombin produced at the end of the 5 min. simulation. (C) The lag time, which is the time to 10% of the maximum cumulative thrombin production for 100% FVIII. (D) The velocity, which is the slope of the cumulative thrombin curve from the lag time to the end of the simulation. Each data point (•) represents a single simulation. The lines are extrapolations between simulation data points.
(EPS)

Figure S3 **The relative roles of intrinsic and extrinsic tenase on Xa generation in response to rFVIIa treatment.** The total cumulative Xa production for different plasma levels of FVIIa (A) and the relative contribution from intrinsic (FVIIIa∶IXa) tenase (B) and extrinsic (TF∶FVIIa) tenase (C). Xa production is normalized by the total cumulative production of Xa for 100% FVIII and 0.1 nM FVIIa.
(EPS)

Text S1 **Computational model details including numerical methods, model equations, and kinetic rate constant and physical properties.**
(PDF)

Video S1 **Platelet (blue) and fibrin (green) accumulation from a normal control on collagen-TF surface at 100 s^−1^.** The direction of flow is from top to bottom.
(AVI)

Video S2 **Platelet (blue) and fibrin (green) accumulation from an individual with mild hemophilia (FVIII∶C = 11.1%) on collagen-TF surface at 100 s^−1^.** The direction of flow is from top to bottom.
(AVI)

Video S3 **Platelet (blue) and fibrin (green) accumulation from an individual with moderate hemophilia (FVIII∶C = 3.1%) on collagen-TF surface at 100 s^−1^.** The direction of flow is from top to bottom.
(AVI)

Video S4 **Platelet (blue) and fibrin (green) accumulation from an individual with severe hemophilia (FVIII∶C = 0.4%) on collagen-TF surface at 100 s^−1^.** The direction of flow is from top to bottom.
(AVI)
