# Supplementary material for: The Effect of Factor VIII Deficiencies and Replacement and Bypass Therapies on Thrombus Formation under Venous Flow Conditions in Microfluidic and Computational Models
Source: PLoS One. 2013 Nov 13;8(11):e78732. doi: 10.1371/journal.pone.0078732 (PMC3827262; doi:10.1371/journal.pone.0078732)
Supplement: Text S1 — Computational model details including numerical methods, model equations, and kinetic rate constant and physical properties. (PDF) [file pone.0078732.s004.pdf]

# Supplemental Computational Model Details

## Overview

Platelets are tracked in four groups: mobile and unactivated, mobile and activated, bound and activated, and bound directly to exposed subendothelium and activated. Mobile, unactivated platelets may bind directly to the subendothelium. Mobile activated platelets may bind either to the subendothelium directly or to other bound platelets. Platelets are modeled as continua, that is, platelet densities rather than discrete objects are tracked for the four populations of platelets just described. Chemicals are tracked in three groups: fluid-phase (advect and diffuse), platelet-bound (immobile), and subendothelium-bound (immobile). Motion of the fluid (blood plasma) is governed by the two-dimensional Navier-Stokes equations with an additional force term that represents the frictional resistance applied to the fluid by the porous thrombus.

## Notation

The coagulation reactions we consider in the computational model are listed in Tables 1-8. $Z_i$ and $E_i$ refer to zymogen $i$ and enzyme $i$ in solution. Superscripts indicate membrane-bound versions of these proteins (e.g., $E_7^{se}$ refers to the TF:VIIa complex and $E_5^m$ refers to Factor Va bound to the platelet surface). Concentrations are denoted in a similar way but with lower-case $z$ and $e$. $P_2, P_5, P_{10}, P_8$ and $P_9$ refer to the binding sites on bound platelets for prothrombin/thrombin, factors V/Va, X/Xa, VIII/VIIIa, and IX/IXa; $[P_i] = N_i^b P^{b,a} + N_i^s P^{se,a}$ refers to the concentrations of these binding sites where $N_2^b$, $N_5^b$, $N_{10}^b$, $N_8^b$, and $N_9^b$ refer to the number of binding sites for the zymogen-enzyme pairs expressed on the surface of a bound platelet (similar notation ${N_i}^s$ describes these quantities for a platelet bound directly to the subendothelium). A complex of $Z_i$ and $E_j$ is denoted $Z_i : E_j$ and its concentration is denoted $[Z_i : E_j]$. Special symbols are used for the platelet-bound 'tenase' VIIIa:IXa and 'prothrombinase' Va:Xa complexes, $TEN$ = VIIIa:IXa and $PRO$= Va:Xa, and $[TEN]$ and $[PRO]$ denote their respective concentrations. The special symbol $TFPIa$ is used for the fluid-phase complex TFPI:Xa, and $[TFPIa]$ denotes its concentration. The inhibitors are denoted $APC$ and $TFPI$ and their concentrations are denoted $[APC]$ and $[TFPI]$. $P_{\text{avail}}$ in the Table 6 is simply the quantity $P_{max} - P^{se,a}$.

## Numerical Methods

We solve the model equations in a rectangular spatial region $R = [0, x_{max}] \times [0, y_{max}]$. For all of the variables, including the fluid, we use a uniform mesh placed over $R$ with equal mesh spacing in both the $x$ and $y$ directions. For each differential equation, we use a finite-difference approximation defined at points on this mesh. During each timestep of the computation, we perform the following series of updates for the unknowns:

1. The discretized Navier-Stokes equations with Brinkman term are solved using a second-order projection method, details of which can be found in (Leiderman, Miller & Fogelson 2008), to give new fluid velocities $u, v$, and pressure, $p$.

2. Platelets activated within the previous timestep are counted and the ADP release function, $\sigma_{release}$, is updated (see (Leiderman & Fogelson 2011) for details about this function).

3. Mobile platelets and fluid-phase chemical concentrations are updated to account for advection using LeVeque's high-resolution advection algorithm (LeVeque 1996).

4. Mobile platelet and fluid-phase chemical concentrations are updated to account for diffusion using a Crank-Nicolson time discretization and a spatial-difference approximation to the spatially-varying diffusion operator.

5. All species are updated to account for reactions using a second-order Runge-Kutta solver.

6. The platelet fractions of bound platelets, $\phi^B$ and $\phi^T$, are calculated and $\alpha(\phi^B)$ and $W(\phi^T)$ are updated.

These steps are repeated until the prescribed final time for the simulation.

## Model Equations for Chemicals

### Subendothelium-bound

$$\frac{\partial z_7^{se}}{\partial t} = k_7^{\text{on}} z_7([TF] - e_7^{setot} - z_7^{setot}) - k_7^{\text{off}} z_7^{se} - k_{z7se:e10}^{+} z_7^{se} e_{10} + k_{z7se:e10}^{-}[Z_7^{se} : E_{10}] - k_{z7se:e2}^{+} z_7^{se} e_2 + k_{z7se:e2}^{-}[Z_7^{se} : E_2] - k_{adh}(\mathbf{x}) z_7^{se}(P^{m,a} + P^{m,u} + P^{b,a}) \quad (1)$$

$$\frac{\partial e_7^{se}}{\partial t} = k_7^{\text{on}} e_7([TF] - e_7^{setot} - z_7^{setot}) - k_7^{\text{off}} e_7^{se} + k_{z7se:e10}^{\text{cat}}[Z_7^{se} : E_{10}] + k_{z7se:e2}^{\text{cat}}[Z_7^{se} : E_2] + (k_{z10:e7se}^{-} + k_{z10:e7se}^{\text{cat}})[Z_{10} : E_7^{se}] - k_{z10:e7se}^{+} z_{10} e_7^{se} + (k_{z9:e7se}^{-} + k_{z9:e7se}^{\text{cat}})[Z_9 : E_7^{se}] - k_{z9:e7se}^{+} z_9 e_7^{se} - k_{tfpia:e7se}^{+}[TFPIa] e_7^{se} + k_{tfpia:e7se}^{-}[TFPIa : E_7^{se}] - k_{adh}(\mathbf{x}) e_7^{se}(P^{m,a} + P^{m,u} + P^{b,a}) \quad (2)$$

$$\frac{\partial [Z_7^{se} : E_2]}{\partial t} = k_{z7se:e2}^{+} z_7^{se} e_2 - (k_{z7se:e2}^{-} + k_{z7se:e2}^{\text{cat}})[Z_7^{se} : E_2] - k_{adh}(\mathbf{x})[Z_7^{se} : E_2](P^{m,a} + P^{m,u} + P^{b,a}) \quad (3)$$

$$\frac{\partial [Z_7^{se} : E_{10}]}{\partial t} = k_{z7se:e10}^{+} z_7^{se} e_{10} - (k_{z7se:e10}^{-} + k_{z7se:e10}^{\text{cat}})[Z_7^{se} : E_{10}] - k_{adh}(\mathbf{x})[Z_7^{se} : E_{10}](P^{m,a} + P^{m,u} + P^{b,a}) \quad (4)$$

$$\frac{\partial [Z_9 : E_7^{se}]}{\partial t} = k_{z9:e7se}^{+} z_9 e_7^{se} - (k_{z9:e7se}^{-} + k_{z9:e7se}^{\text{cat}})[Z_9 : E_7^{se}] - k_{adh}(\mathbf{x})[Z_9 : E_7^{se}](P^{m,a} + P^{m,u} + P^{b,a}) \quad (5)$$

$$\frac{\partial [Z_{10} : E_7^{se}]}{\partial t} = k_{z10:e7se}^{+} z_{10} e_7^{se} - (k_{z10:e7se}^{-} + k_{z10:e7se}^{\text{cat}})[Z_{10} : E_7^{se}] - k_{adh}(\mathbf{x})[Z_{10} : E_7^{se}](P^{m,a} + P^{m,u} + P^{b,a}) \quad (6)$$

$$\frac{\partial [TFPIa : E_7^{se}]}{\partial t} = -k_{tfpia:e7se}^{-}[TFPIa : E_7^{se}] + k_{tfpia:e7se}^{+}[TFPIa] e_7^{se} - k_{adh}(\mathbf{x})[TFPIa : E_7^{se}](P^{m,a} + P^{m,u} + P^{b,a}) \quad (7)$$

$$\frac{\partial [TF]}{\partial t} = -k_{adh}(\mathbf{x})[TF](P^{m,a} + P^{m,u} + P^{b,a}) \quad (8)$$

**Platelet-bound**

$$\begin{aligned}\frac{\partial z_2^m}{\partial t} &= k_2^{\text{on}} z_2 (N_2^b P^{b,a} + N_2^{se} P^{se,a} - z_2^{m\,tot} - e_2^{m\,tot}) - k_2^{\text{off}} z_2^m \\ &\quad - k^+_{z2m:pro} z_2^m [PRO] + k^-_{z2m:pro} [Z_2^m : PRO] \end{aligned} \quad (9)$$

$$\begin{aligned}\frac{\partial e_2^m}{\partial t} &= k_2^{\text{on}} e_2 (N_2^b P^{b,a} + N_2^{se} P^{se,a} - z_2^{m\,tot} - e_2^{m\,tot}) \quad (10) \\ &\quad - k_2^{\text{off}} e_2^m + k^{\text{cat}}_{z2m:pro} [Z_2^m : PRO] \\ &\quad + (k^{\text{cat}}_{z5m:e2m} + k^-_{z5m:e2m}) [Z_5^m : E_2^m] - k^+_{z5m:e2m} z_5^m e_2^m \\ &\quad + (k^{\text{cat}}_{z8m:e2m} + k^-_{z8m:e2m}) [Z_8^m : E_2^m] - k^+_{z8m:e2m} z_8^m e_2^m \end{aligned}$$

$$\begin{aligned}\frac{\partial z_5^m}{\partial t} &= k_5^{\text{on}} z_5 (N_5^b P^{b,a} + N_5^{se} P^{se,a} - z_5^{m\,tot} - e_5^{m\,tot}) - k_5^{\text{off}} z_5^m \quad (11) \\ &\quad - k^+_{z5m:e10m} z_5^m e_{10}^m + k^-_{z5m:e10m} [Z_5^m : E_{10}^m] \\ &\quad - k^+_{z5m:e2m} z_5^m e_2^m + k^-_{z5m:e2m} [Z_5^m : E_2^m] \end{aligned}$$

$$\begin{aligned}\frac{\partial e_5^m}{\partial t} &= k_5^{\text{on}} e_5 (N_5^b P^{b,a} + N_5^{se} P^{se,a} - z_5^{m\,tot} - e_5^{m\,tot}) - k_5^{\text{off}} e_5^m \quad (12) \\ &\quad + k^{\text{cat}}_{z5m:e10m} [Z_5^m : E_{10}^m] + k^{\text{cat}}_{z5m:e2m} [Z_5^m : E_2^m] + k^-_{\text{pro}} [PRO] \quad (13) \\ &\quad - k^+_{\text{pro}} e_5^m e_{10}^m - k^+_{apc:e5m} [APC] e_5^m + k^-_{apc:e5m} [APC : E_5^m] \end{aligned}$$

$$\begin{aligned}\frac{\partial z_8^m}{\partial t} &= k_8^{\text{on}} z_8 (N_8^b P^{b,a} + N_8^{se} P^{se,a} - z_8^{m\,tot} - e_8^{m\,tot}) - k_8^{\text{off}} z_8^m \quad (14) \\ &\quad - k^+_{z8m:e10m} z_8^m e_{10}^m + k^-_{z8m:e10m} [Z_8^m : E_{10}^m] \\ &\quad - k^+_{z8m:e2m} z_8^m e_2^m + k^-_{z8m:e2m} [Z_8^m : E_2^m] \end{aligned}$$

$$\begin{aligned}\frac{\partial e_8^m}{\partial t} &= k_8^{\text{on}} e_8 (N_8^b P^{b,a} + N_8^{se} P^{se,a} - z_8^{m\,tot} - e_8^{m\,tot}) - k_8^{\text{off}} e_8^m \quad (15) \\ &\quad + k^{\text{cat}}_{z8m:e10m} [Z_8^m : E_{10}^m] + k^{\text{cat}}_{z8m:e2m} [Z_8^m : E_2^m] + k^-_{\text{ten}} [TEN] \\ &\quad - k^+_{\text{ten}} e_8^m e_9^m - k^+_{apc:e8m} [APC] e_8^m + k^-_{apc:e8m} [APC : E_8^m] \end{aligned}$$

$$\frac{\partial z_9^m}{\partial t} = k_9^{\text{on}} z_9 (N_9^b P^{b,a} + N_9^{se} P^{se,a} - z_9^{m\,tot} - e_9^{m\,tot}) - k_9^{\text{off}} z_9^m \quad (16)$$

$$\begin{aligned}\frac{\partial e_9^m}{\partial t} &= k_9^{\text{on}} e_9 (N_9^b P^{b,a} + N_9^{se} P^{se,a} - z_9^{m\,tot} - e_9^{m\,tot}) - k_9^{\text{off}} e_9^m \quad (17) \\ &\quad + k^-_{\text{ten}} [TEN] - k^+_{\text{ten}} e_8^m e_9^m \end{aligned}$$

$$\begin{aligned}\frac{\partial z_{10}^m}{\partial t} &= k_{10}^{\text{on}} z_{10} (N_{10}^b P^{b,a} + N_{10}^{se} P^{se,a} - e_{10}^{m\,tot} - z_{10}^{m\,tot}) - k_{10}^{\text{off}} z_{10}^m \quad (18) \\ &\quad + k^-_{z10m:ten} [Z_{10}^m : TEN] - k^+_{z10m:ten} z_{10}^m [TEN] \\ &\quad + k^-_{z10m:ten} [Z_{10}^m : TEN^*] - k^+_{z10m:ten} z_{10}^m [TEN^*] \end{aligned}$$

$$\begin{aligned}\frac{\partial e_{10}^m}{\partial t} &= k_{10}^{\text{on}} e_{10}(N_{10}^b P^{b,a} + N_{10}^{se} P^{se,a} - e_{10}^{m\,tot} - z_{10}^{m\,tot}) - k_{10}^{\text{off}} e_{10}^m \\ &+(k_{z5m:e10m}^- + k_{z5m:e10m}^{\text{cat}})[Z_5^m : E_{10}^m] - k_{z5m:e10m}^+ z_5^m e_{10}^m \\ &+(k_{z8m:e10m}^- + k_{z8m:e10m}^{\text{cat}})[Z_8^m : E_{10}^m] - k_{z8m:e10m}^+ z_8^m e_{10}^m \\ &+k_{\text{pro}}^-[PRO] - k_{\text{pro}}^+ e_5^m e_{10}^m + k_{z10m:ten}^{\text{cat}}[Z_{10}^m : TEN] \\ &+k_{z10m:ten}^{\text{cat}}[Z_{10}^m : TEN^*]\end{aligned} \tag{19}$$

$$\begin{aligned}\frac{\partial [TEN]}{\partial t} &= k_{\text{ten}}^+ e_8^m e_9^m - k_{\text{ten}}^-[TEN] - k_{z10m:ten}^+ z_{10}^m [TEN] \\ &+(k_{z10m:ten}^{\text{cat}} + k_{z10m:ten}^-)[Z_{10}^m : TEN]\end{aligned} \tag{20}$$

$$\begin{aligned}\frac{\partial [PRO]}{\partial t} &= k_{\text{pro}}^+ e_5^m e_{10}^m - k_{\text{pro}}^-[PRO] - k_{z2m:pro}^+ z_2^m [PRO] \\ &+(k_{z2m:pro}^{\text{cat}} + k_{z2m:pro}^-)[Z_2^m : PRO]\end{aligned} \tag{21}$$

$$\frac{\partial [Z_2^m : PRO]}{\partial t} = k_{z2m:pro}^+ z_2^m [PRO] - (k_{z2m:pro}^- + k_{z2m:pro}^{\text{cat}})[Z_2^m : PRO] \tag{22}$$

$$\frac{\partial [Z_5^m : E_2^m]}{\partial t} = k_{z5m:e2m}^+ z_5^m e_2^m - (k_{z5m:e2m}^- + k_{z5m:e2m}^{\text{cat}})[Z_5^m : E_2^m] \tag{23}$$

$$\frac{\partial [Z_5^m : E_{10}^m]}{\partial t} = k_{z5m:e10m}^+ z_5^m e_{10}^m - (k_{z5m:e10m}^- + k_{z5m:e10m}^{\text{cat}})[Z_5^m : E_{10}^m] \tag{24}$$

$$\frac{\partial [Z_8^m : E_2^m]}{\partial t} = k_{z8m:e2m}^+ z_8^m e_2^m - (k_{z8m:e2m}^- + k_{z8m:e2m}^{\text{cat}})[Z_8^m : E_2^m] \tag{25}$$

$$\frac{\partial [Z_8^m : E_{10}^m]}{\partial t} = k_{z8m:e10m}^+ z_8^m e_{10}^m - (k_{z8m:e10m}^- + k_{z8m:e10m}^{\text{cat}})[Z_8^m : E_{10}^m] \tag{26}$$

$$\frac{\partial [Z_{10}^m : TEN]}{\partial t} = k_{z10m:ten}^+ z_{10}^m [TEN] - (k_{z10m:ten}^- + k_{z10m:ten}^{\text{cat}})[Z_{10}^m : TEN] \tag{27}$$

$$\frac{\partial [APC : E_5^m]}{\partial t} = k_{apc:e5m}^+ [APC] e_5^m - (k_{apc:e5m}^- + k_{apc:e5m}^{\text{cat}})[APC : E_5^m] \tag{28}$$

$$\frac{\partial [APC : E_8^m]}{\partial t} = k_{apc:e8m}^+ [APC] e_8^m - (k_{apc:e8m}^- + k_{apc:e8m}^{\text{cat}})[APC : E_8^m] \tag{29}$$

$$\begin{aligned}\frac{\partial e_9^{m,*}}{\partial t} &= k_9^{on} e_9^m (N_{9*}^b P^{b,a} + N_{9*}^{se} P^{se,a} - e_9^{m,*} - [TEN^*] - [Z_{10}^m : TEN^*]) \\ &-k_9^{off} e_9^{m,*} + k_{ten}^-[TEN^*] - k_{ten}^+ e_8^m e_9^{m,*}\end{aligned} \tag{30}$$

$$\begin{aligned}\frac{\partial [TEN^*]}{\partial t} &= k_{ten}^+ e_8^m e_9^{m,*} - k_{ten}^-[TEN^*] \\ &+(k_{z10m:ten}^- + k_{z10m:ten}^{\text{cat}})[Z_{10}^m : TEN^*] - k_{z10m:ten}^+ z_{10}^m [TEN^*]\end{aligned} \tag{31}$$

$$\frac{\partial [Z_{10}^m : TEN^*]}{\partial t} = k_{z10m:ten}^+ z_{10}^m [TEN^*] - (k_{z10m:ten}^- + k_{z10m:ten}^{\text{cat}})[Z_{10}^m : TEN^*] \tag{32}$$

**Fluid-phase**

$$\frac{\partial z_2}{\partial t} = -\nabla\cdot(\mathbf{u}z_2 - D\nabla z_2) - k_2^{\text{on}} z_2(N_2^b P^{b,a} + N_2^{se}P^{se,a} - z_2^{mtot} - e_2^{mtot}) + k_2^{\text{off}} z_2^m \tag{33}$$

$$\begin{aligned}\frac{\partial e_2}{\partial t} = & -\nabla\cdot(\mathbf{u}e_2 - D\nabla e_2) - k_2^{\text{on}} e_2(N_2^b P^{b,a} + N_2^{se}P^{se,a} - z_2^{mtot} - e_2^{mtot}) + k_2^{\text{off}} e_2^m \\ & +(k_{z5:e2}^{\text{cat}} + k_{z5:e2}^{-})[Z_5:E_2] - k_{z5:e2}^{+} z_5 e_2 \\ & +(k_{z7:e2}^{-} + k_{z7:e2}^{\text{cat}})[Z_7:E_2] - k_{z7:e2}^{+} z_7 e_2 \\ & +(k_{z8:e2}^{\text{cat}} + k_{z8:e2}^{-})[Z_8:E_2] - k_{z8:e2}^{+} z_8 e_2 - k_2^{\text{in}} e_2\end{aligned} \tag{34}$$

$$\begin{aligned}\frac{\partial z_5}{\partial t} = & -\nabla\cdot(\mathbf{u}z_5 - D\nabla z_5) - k_5^{\text{on}} z_5(N_5^b P^{b,a} + N_5^{se}P^{se,a} - z_5^{mtot} - e_5^{mtot}) + k_5^{\text{off}} z_5^m \\ & -k_{z5:e2}^{+} z_5 e_2 + k_{z5:e2}^{-}[Z_5:E_2] + N_5 \frac{\partial(P^{b,a} + P^{se,a})}{\partial t}\end{aligned} \tag{35}$$

$$\begin{aligned}\frac{\partial e_5}{\partial t} = & -\nabla\cdot(\mathbf{u}e_5 - D\nabla e_5) - k_5^{\text{on}} e_5(N_5^b P^{b,a} + N_5^{se}P^{se,a} - z_5^{mtot} - e_5^{mtot}) + k_5^{\text{off}} e_5^m \\ & +k_{z5:e2}^{\text{cat}}[Z_5:E_2]\end{aligned} \tag{36}$$

$$\begin{aligned}\frac{\partial z_7}{\partial t} = & -\nabla\cdot(\mathbf{u}z_7 - D\nabla z_7) - k_{z7:e10}^{+} z_7 e_{10} + k_{z7:e10}^{-}[Z_7:E_{10}] + k_{z7:e2}^{-}[Z_7:E_2] \\ & -k_{z7:e2}^{+} z_7 e_2\end{aligned} \tag{37}$$

$$\frac{\partial e_7}{\partial t} = -\nabla\cdot(\mathbf{u}e_7 - D\nabla e_7) + k_{z7:e10}^{\text{cat}}[Z_7:E_{10}] + k_{z7:e2}^{\text{cat}}[Z_7:E_2]$$

$$\begin{aligned}\frac{\partial z_8}{\partial t} = & -\nabla\cdot(\mathbf{u}z_8 - D\nabla z_8) - k_8^{\text{on}} z_8(N_8^b P^{b,a} + N_8^{se}P^{se,a} - z_8^{mtot} - e_8^{mtot}) + k_8^{\text{off}} z_8^m \\ & -k_{z8:e2}^{+} z_8 e_2 + k_{z8:e2}^{-}[Z_8:E_2]\end{aligned} \tag{38}$$

$$\begin{aligned}\frac{\partial e_8}{\partial t} = & -\nabla\cdot(\mathbf{u}e_8 - D\nabla e_8) - k_8^{\text{on}} e_8(N_8^b P^{b,a} + N_8^{se}P^{se,a} - z_8^{mtot} - e_8^{mtot}) + k_8^{\text{off}} e_8^m \\ & +k_{z8:e2}^{\text{cat}}[Z_8:E_2]\end{aligned} \tag{39}$$

$$\frac{\partial z_9}{\partial t} = -\nabla\cdot(\mathbf{u}z_9 - D\nabla z_9) - k_9^{\text{on}} z_9(N_9^b P^{b,a} + N_9^{se}P^{se,a} - z_9^{mtot} - e_9^{mtot}) + k_9^{\text{off}} z_9^m \tag{40}$$

$$\frac{\partial e_9}{\partial t} = -\nabla\cdot(\mathbf{u}e_9 - D\nabla e_9) - k_9^{\text{on}} e_9(N_9^b P^{b,a} + N_9^{se}P^{se,a} - z_9^{mtot} - e_9^{mtot}) + k_9^{\text{off}} e_9^m \tag{41}$$

$$\begin{aligned}& -k_9^{\text{in}} e_9 - k_9^{on} e_9^m(N_{9*}^b P^{b,a} + N_{9*}^{se}P^{se,a} - e_9^{m,*} - [TEN^*] - [Z_{10}^m : TEN^*]) \\ & +k_9^{off} e_9^{m,*}\end{aligned} \tag{42}$$

$$\frac{\partial z_{10}}{\partial t} = -\nabla \cdot (\mathbf{u} z_{10} - D\nabla z_{10}) - k_{10}^{\text{on}} z_{10}(N_{10}^{b} P^{b,a} + N_{10}^{se} P^{se,a} - e_{10}^{m\,tot} - z_{10}^{m\,tot}) + k_{10}^{\text{off}} z_{10}^{m} \quad (43)$$

$$\frac{\partial e_{10}}{\partial t} = -\nabla \cdot (\mathbf{u} e_{10} - D\nabla e_{10}) - k_{10}^{\text{on}} e_{10}(N_{10}^{b} P^{b,a} + N_{10}^{se} P^{se,a} - e_{10}^{m\,tot} - z_{10}^{m\,tot}) + k_{10}^{\text{off}} e_{10}^{m} - k_{tfpia:e10}^{+}[TFPI]e_{10} + k_{tfpia:e10}^{-}[TFPIa] + (k_{z7:e10}^{-} + k_{z7:e10}^{\text{cat}})[Z_7 : E_{10}] - k_{z7:e10}^{+} z_7 e_{10} - k_{10}^{\text{in}} e_{10} \quad (44)$$

$$\frac{\partial [Z_5 : E_2]}{\partial t} = -\nabla \cdot (\mathbf{u}[Z_5 : E_2] - D\nabla[Z_5 : E_2]) + k_{z5:e2}^{+} z_5 e_2 - (k_{z5:e2}^{-} + k_{z5:e2}^{\text{cat}})[Z_5 : E_2] \quad (45)$$

$$\frac{\partial [Z_7 : E_2]}{\partial t} = -\nabla \cdot (\mathbf{u}[Z_7 : E_2] - D\nabla[Z_7 : E_2]) + k_{z7:e2}^{+} z_7 e_2 - (k_{z7:e2}^{-} + k_{z7:e2}^{\text{cat}})[Z_7 : E_2] \quad (46)$$

$$\frac{\partial [Z_7 : E_{10}]}{\partial t} = -\nabla \cdot (\mathbf{u}[Z_7 : E_{10}] - D\nabla[Z_7 : E_{10}]) + k_{z7:e10}^{+} z_7 e_{10} - (k_{z7:e10}^{-} + k_{z7:e10}^{\text{cat}})[Z_7 : E_{10}] \quad (47)$$

$$\frac{\partial [Z_8 : E_2]}{\partial t} = -\nabla \cdot (\mathbf{u}[Z_8 : E_2] - D\nabla[Z_8 : E_2]) + k_{z8:e2}^{+} z_8 e_2 - (k_{z8:e2}^{-} + k_{z8:e2}^{\text{cat}})[Z_8 : E_2] \quad (48)$$

$$\frac{\partial [APC]}{\partial t} = -\nabla \cdot (\mathbf{u}[APC] - D\nabla[APC]) - k_{apc:e5m}^{+}[APC]e_5^{m} + (k_{apc:e5m}^{-} + k_{apc:e5m}^{\text{cat}})[APC : E_5^{m}] - k_{apc:e8m}^{+}[APC]e_8^{m} + (k_{apc:e8m}^{-} + k_{apc:e8m}^{\text{cat}})[APC : E_8^{m}] \quad (49)$$

$$\frac{\partial [TFPI]}{\partial t} = -\nabla \cdot (\mathbf{u}[TFPI] - D\nabla[TFPI]) - k_{tfpia:e10}^{+}[TFPI]e_{10} + k_{tfpia:e10}^{-}[TFPIa] \quad (50)$$

$$\frac{\partial [TFPIa]}{\partial t} = -\nabla \cdot (\mathbf{u}[TFPIa] - D\nabla[TFPIa]) + k_{tfpia:e10}^{+}[TFPI]e_{10} - k_{tfpia:e10}^{-}[TFPIa] \quad (51)$$

$$\frac{\partial [ADP]}{\partial t} = -\mathbf{u} \cdot \nabla[ADP] + \nabla \cdot (D[\nabla ADP]) + \sigma_{release} \quad (52)$$

**Subendothelium Boundary Conditions**

$$-D\frac{\partial e_2}{\partial y} = -k^+_{z7se:e2} z_7^{se} e_2 + (k^-_{z7se:e2} + k^{\mathrm{cat}}_{z7se:e2})[Z_7^{se} : E_2] \quad (53)$$

$$-D\frac{\partial z_7}{\partial y} = -k_7^{on} z_7([TF] - z_7^{se,tot} - e_7^{se,tot}) + k_7^{off} z_7^{se} \quad (54)$$

$$-D\frac{\partial e_7}{\partial y} = -k_7^{on} e_7([TF] - z_7^{se,tot} - e_7^{se,tot}) + k_7^{off} e_7^{se} \quad (55)$$

$$-D\frac{\partial z_9}{\partial y} = -k^+_{z9:e7se} z_9 e_7^{se} + k^-_{z9:e7se}[Z_9 : E_7^{se}] \quad (56)$$

$$-D\frac{\partial e_9}{\partial y} = k^{\mathrm{cat}}_{z9:e7se}[Z_9 : E_7^{se}] \quad (57)$$

$$-D\frac{\partial z_{10}}{\partial y} = -k^+_{z10:e7se} z_{10} e_7^{se} + k^-_{z10:e7se}[Z_{10} : E_7^{se}] \quad (58)$$

$$-D\frac{\partial e_{10}}{\partial y} = -k^+_{z7se:e10} e_{10} z_7^{se} + k^{\mathrm{cat}}_{z10:e7se}[Z_{10} : E_7^{se}] \quad (59)$$

$$+(k^-_{z7se:e10} + k^{\mathrm{cat}}_{z7se:e10})[Z_7^{se} : E_{10}]$$

$$-D\frac{\partial [TFPIa]}{\partial y} = -k^+_{tfpia:e7se}[TFPIa] e_7^{se} + k^-_{tfpia:e7se}[TFPIa : E_7^{se}] \quad (60)$$

## Kinetic and Physical Parameters

| | | |
|---|---|---|
| Platelets | $2.5\times10^{-7}$ cm$^2$/s | a |
| ADP | $5\times10^{-6}$ cm$^2$/s | b |
| All Other Chemical Species | $5\times10^{-7}$ cm$^2$/s | c |

Table 1: DIFFUSION COEFFICIENTS FOR PLATELETS AND MOBILE CHEMICAL SPECIES (a) From (Turitto & Leonard 1972). (b) From (Grabowski et al. 1978). (c) From (Young et al. 1980).

| | | |
|---|---|---|
| PROTHROMBIN | 1.4 $\mu$M | a |
| FACTOR V | 0.01 $\mu$M | b |
| FACTOR VII | 0.01 $\mu$M | a |
| FACTOR VIIa | 0.1 nM | c |
| FACTOR VIII | 1.0 nM | a |
| FACTOR IX | 0.09 $\mu$M | a |
| FACTOR X | 0.17 $\mu$M | a |
| TFPI | 2.5 nM | d |
| PLATELETS, $P_0$ | $2.5(10)^5$/mm$^3$ | e |
| $N_2^b, N_2^{se}$ | 2000 | f |
| $N_5^b, N_5^{se}$ | 3000 | g |
| $N_8^b, N_8^{se}$ | 450 | h |
| $N_9^b, N_9^{se}$ | 500 | i |
| $N_{10}^b, N_{10}^{se}$ | 2700 | j |

Table 2: NORMAL CONCENTRATIONS AND SURFACE BINDING SITE NUMBERS (a) From (Mann et al. 1990). (b) From (Mann et al. 1991). (c) (Morrissey 1995) suggests that normal plasma concentration of fVIIa is about 1% of the normal fVII concentration. (d) From (Novotny et al. 1991). (e) From (Weiss 1975). (f) From (Brass et al. 1994). (g) From (Walsh 1994). (h) From (Nesheim et al. 1988). (i) From (Ahmad et al. 1989). (j) From (Mann et al. 1992).

| Reaction | Reactants | Complex | Product | $M^{-1}sec^{-1}$ | $sec^{-1}$ | $sec^{-1}$ | Note |
|---|---|---|---|---|---|---|---|
| Activation (of-,by-) | | | | | | | |
| (TF:VII,Xa) | $E_{10}$, $Z_7^{se}$ | $Z_7^{se} : E_{10}$ | $E_7^{se}$ | $k^+_{z7se:e10}$=$5.0 \cdot 10^6$ | $k^-_{z7se:e10}$=1.0 | $k^{cat}_{z7se:e10}$=5.0 | a |
| (TF:VII,IIa) | $E_2$, $Z_7^{se}$ | $Z_7^{se} : E_2$ | $E_7^{se}$ | $k^+_{z7se:e2}$=$3.92 \cdot 10^5$ | $k^-_{z7se:e2}$=1.0 | $k^{cat}_{z7se:e2}$=$6.1 \cdot 10^{-2}$ | b |
| (X,TF:VIIa) | $E_7^{se}$, $Z_{10}$ | $Z_{10} : E_7^{se}$ | $E_{10}$ | $k^+_{z10:e7se}$=$8.95 \cdot 10^6$ | $k^-_{z10:e7se}$=1.0 | $k^{cat}_{z10:e7se}$=1.15 | c |
| (IX,TF:VIIa) | $E_7^{se}$, $Z_9$ | $Z_9 : E_7^{se}$ | $E_9$ | $k^+_{z9:e7se}$=$8.95 \cdot 10^6$ | $k^-_{z9:e7se}$=1.0 | $k^{cat}_{z9:e7se}$=1.15 | d |
| Binding (-,with -) | | | | | | | |
| (VII,TF) | $Z_7$, $TF$ | | $Z_7^{se}$ | $k_7^{on}$=$5.0 \cdot 10^7$ | $k_7^{off}$=$5.0 \cdot 10^{-3}$ | | e |
| (VIIa,TF) | $E_7$, $TF$ | | $E_7^{se}$ | $k_7^{on}$=$5.0 \cdot 10^7$ | $k_7^{off}$=$5.0 \cdot 10^{-3}$ | | e |

Table 3: REACTIONS ON SUBENDOTHELIUM (a) $k^{cat}_{z7se:e10} = 5.0$ $sec^{-1}$ and $K_M = 1.2 \cdot 10^{-6}$ M (Butenas & Mann 1996). (b) $k^{cat}_{z7se:e2} = 6.1 \cdot 10^{-2}$ $sec^{-1}$ and $K_M = 2.7 \cdot 10^{-6}$ M (Butenas & Mann 1996). (d) $k^{cat}_{z10:e7se} = 1.15$ $sec^{-1}$ and $K_M = 4.5 \cdot 10^{-7}$ M (Mann et al. 1990). (d) We assume that the reaction constants for TF:VIIa activation of fIX are the same as for TF:VIIa activation of fX. (e) $K_d = 1.0 \cdot 10^{-10}$ M (Nemerson 1992).

| Reaction | Reactants | Complex | Product | $M^{-1}sec^{-1}$ | $sec^{-1}$ | $sec^{-1}$ | Note |
|---|---|---|---|---|---|---|---|
| Activation (of-,by-) | | | | | | | |
| (VII,Xa) | $Z_7$, $E_{10}$ | $Z_7 : E_{10}$ | $E_7$ | $k^{+}_{z7:e10}$=5 $\cdot 10^6$ | $k^{-}_{z7:e10}$=1.0 | $k^{cat}_{z7:e10}$=5.0 | a |
| (VII,IIa) | $Z_7$, $E_2$ | $Z_7 : E_2$ | $E_7$ | $k^{+}_{z7:e2}$=3.92 $\cdot 10^5$ | $k^{-}_{z7:e2}$=1.0 | $k^{cat}_{z7:e2}$=6.1 $\cdot 10^{-2}$ | b |
| (V,IIa) | $Z_5$, $E_2$ | $Z_5 : E_2$ | $E_5$ | $k^{+}_{z5:e2}$= 1.73 $\cdot 10^7$ | $k^{-}_{z5:e2}$=1.0 | $k^{cat}_{z5:e2}$=0.23 | c |
| (VIII,IIa) | $Z_8$, $E_2$ | $Z_8 : E_2$ | $E_8$ | $k^{+}_{z8:e2}$=2.64 $\cdot 10^7$ | $k^{-}_{z8:e2}$=1.0 | $k^{cat}_{z8:e2}$=0.9 | d |

Table 4: REACTIONS IN THE PLASMA (a) $k^{cat}_{z7:e10} = 5.0$ sec$^{-1}$ and $K_M = 1.2 \cdot 10^{-6}$ M (Butenas & Mann 1996). (b) $k^{cat}_{z7:e2} = 6.1 \cdot 10^{-2}$ sec$^{-1}$ and $K_M = 2.7 \cdot 10^{-6}$ M (Butenas & Mann 1996) (c) $k^{cat}_{z5:e2} = 0.23$ sec$^{-1}$ and $K_M = 7.17 \cdot 10^{-8}$ M (Monkovic & Tracy 1990*b*). (d) $k^{cat}_{z8:e2} = 0.9$ sec$^{-1}$ (Hill-Eubanks & Lollar 1990) and $K_M = 2 \cdot 10^{-7}$ M (Lollar et al. 1985).

| Reaction | Reactants | Products | $M^{-1}sec^{-1}$ | $sec^{-1}$ | Note |
|---|---|---|---|---|---|
| | | | | | |
| Factors IX | $Z_9$, $P_9$ | $Z_9^m$ | $k_9^{on}$=1.0 · $10^7$ | $k_9^{off}$=2.5 · $10^{-2}$ | a |
| Factors IXa | $E_9$, $P_9$ | $E_9^m$ | $k_9^{on}$=1.0 · $10^7$ | $k_9^{off}$=2.5 · $10^{-2}$ | a |
| Factors IXa | $E_9$, $P_9^*$ | $E_9^{m,*}$ | $k_9^{on}$=1.0 · $10^7$ | $k_9^{off}$=2.5 · $10^{-2}$ | b |
| Factors X | $Z_{10}$, $P_{10}$ | $Z_{10}^m$ | $k_{10}^{on}$=1.0 · $10^7$ | $k_{10}^{off}$=2.5 · $10^{-2}$ | a |
| Factors Xa | $E_{10}$, $P_{10}$ | $E_{10}^m$ | $k_{10}^{on}$=1.0 · $10^7$ | $k_{10}^{off}$=2.5 · $10^{-2}$ | a |
| Factors V | $Z_5$, $P_5$ | $Z_5^m$ | $k_5^{on}$=5.7 · $10^7$ | $k_5^{off}$=0.17 | c |
| Factors Va | $E_5$, $P_5$ | $E_5^m$ | $k_5^{on}$=5.7 · $10^7$ | $k_5^{off}$=0.17 | c |
| Factors VIII | $Z_8$, $P_8$ | $Z_8^m$ | $k_8^{on}$=5.0 · $10^7$ | $k_8^{off}$=0.17 | d |
| Factors VIIIa | $E_8$, $P_8$ | $E_8^m$ | $k_8^{on}$=5.0 · $10^7$ | $k_8^{off}$=0.17 | d |
| Factors II | $Z_2$, $P_2$ | $Z_2^m$ | $k_2^{on}$=1.0 · $10^7$ | $k_2^{off}$=5.9 | e |
| Factors IIa | $E_2$, $P_2$ | $E_2$ | $k_2^{on}$=1.0 · $10^7$ | $k_2^{off}$=5.9 | e |

Table 5: BINDING TO PLATELET SURFACES (a) For fIX binding to platelets, $K_d = 2.5 \cdot 10^{-9}$ M (Ahmad et al. 1989), and for fX binding to platelets, $K_d$ has approximately the same value (Walsh 1994). For fX binding to PCPS vesicles, the on-rate is about $10^7$ $M^{-1}sec^{-1}$ and the off-rate is about 1.0 $sec^{-1}$ (Krishnaswamy et al. 1988) giving a dissociation constant of about $10^{-7}$ M. To estimate on- and off-rates for the higher-affinity binding of fX to platelets, we keep the on-rate the same as for vesicles and adjust the off-rate to give the correct dissociation constant. The rates for fIX binding with platelets are taken to be the same as for fX binding. (b) We assume binding constants for fIXa binding to the specific fIXa binding sites are the same as for shared sites. (c) fV binds with high-affinity to phospholipids (PCPS) (Krishnaswamy et al. 1988) and we use the same rate constants reported there to describe fV binding to platelets. (d) The $K_d$ for fVIII binding with platelets is taken from (Nesheim et al. 1988). We set the off-rate $k_8^{off}$ for fVIII binding to platelets equal to that for fV binding to platelets, and calculate the on-rate $k_8^{on}$. (e) For prothrombin interactions with platelets, $K_d$ is reported to be $5.9 \cdot 10^{-7}$ M (Mann 1994). We choose $k_2^{off}$ and set $k_2^{on} = k_2^{off}/K_d$.

| Reaction | Reactants | Complex | Product | $M^{-1}sec^{-1}$ | $sec^{-1}$ | $sec^{-1}$ | Note |
|---|---|---|---|---|---|---|---|
| Activation (of-,by-) | | | | | | | |
| (V,Xa) | $Z_5^m$, $E_{10}^m$ | $Z_5^m : E_{10}^m$ | $E_5^m$ | $k^+_{z5m:e10m}$=1.0 · $10^8$ | $k^-_{z5m:e10m}$=1.0 | $k^{cat}_{z5m:e10m}$=4.6 · $10^{-2}$ | a |
| (V,IIa) | $Z_5^m$, $E_2^m$ | $Z_5^m : E_2^m$ | $E_5^m$ | $k^+_{z5m:e2m}$=1.73 · $10^7$ | $k^-_{z5m:e2m}$=1.0 | $k^{cat}_{z5m:e2m}$= 0.23 | b |
| (VIII,Xa) | $Z_8^m$, $E_{10}^m$ | $Z_8^m : E_{10}^m$ | $E_8^m$ | $k^+_{z8m:e10m}$=5.1 · $10^7$ | $k^-_{z8m:e10m}$=1.0 | $k^{cat}_{z8m:e10m}$=2.3 · $10^{-2}$ | c |
| (VIII,IIa) | $Z_8^m$, $E_2^m$ | $Z_8^m : E_2^m$ | $E_8^m$ | $k^+_{z8m:e2m}$=2.64 · $10^7$ | $k^-_{z8m:e2m}$=1.0 | $k^{cat}_{z8m:e2m}$= 0.9 | d |
| (X,VIIIa:IXa) | $Z_{10}^m$, $TEN$ | $Z_{10}^m : TEN$ | $E_{10}^m$ | $k^+_{z10m:ten}$=1.31 · $10^8$ | $k^-_{z10m:ten}$=1.0 | $k^{cat}_{z10m:ten}$=20.0 | f |
| (X,VIIIa:IXa*) | $Z_{10}^m$, $TEN^*$ | $Z_{10}^m : TEN^*$ | $E_{10}^m$ | $k^+_{z10m:ten}$=1.31 · $10^8$ | $k^-_{z10m:ten}$=1.0 | $k^{cat}_{z10m:ten}$=20.0 | f |
| (II,Va:Xa) | $Z_2^m$, $PRO$ | $Z_2^m : PRO$ | $E_2^m$ | $k^+_{z2m:pro}$= 1.03 · $10^8$ | $k^-_{z2m:pro}$=1.0 | $k^{cat}_{z2m:pro}$=30.0 | g |
| Binding (-,with -) | | | | | | | |
| (IIIa,IXa) | $E_8^m$, $E_9^m$ | | $TEN$ | $k^+_{ten}$=1.0 · $10^8$ | $k^-_{ten}$=0.01 | | e |
| (VIIIa,IXa*) | $E_8^m$, $E_9^{m,*}$ | | $TEN^*$ | $k^+_{ten}$= 1.0 · $10^8$ | $k^-_{ten}$=0.01 | | e |
| (Va,Xa) | $E_5^m$, $E_{10}^m$ | | $PRO$ | $k^+_{pro}$=1.0 · $10^8$ | $k^-_{pro}$=0.01 | | e |

Table 6: REACTIONS ON PLATELET SURFACES (a) $k^{cat}_{z5m:e10m} = 0.046$ $sec^{-1}$ and $K_M = 10.4 \cdot 10^{-9}$ M (Monkovic & Tracy 1990*a*). (b) The rate constants for thrombin activation of fV on platelets are assumed to be the same as in plasma. (c) $k^{cat}_{z8m:e10m} = 0.023$ $sec^{-1}$ and $K_M = 2.0 \cdot 10^{-8}$ M (Lollar et al. 1985). (d) The rate constants for thrombin activation of fVIII on platelets are assumed to be the same as in plasma. (e) The formation of the tenase and prothrombinase complexes is assumed to be very fast with $K_d = 1.0 \cdot 10^{-10}$ M (Mann 1987). (f) $k^{cat}_{z10m:ten} = 20$ $sec^{-1}$ and $K_M = 1.6 \cdot 10^{-7}$ M (Rawala-Sheikh et al. 1990). (g) $k^{cat}_{z2m:pro} = 30$ $sec^{-1}$ and $K_M = 3.0 \cdot 10^{-7}$ M (Nesheim et al. 1992).

| Reaction | Reactants | Product | $M^{-1}sec^{-1}$ | $sec^{-1}$ | Note |
|---|---|---|---|---|---|
| Inactivation (of-,by-) | | | | | |
| (IXa,ATIII) | $E_9$ | $E_9,^{in}$ | | $k_9^{\rm in}$=0.1 | a |
| (Xa,ATIII) | $E_{10}$ | $E_{10},^{in}$ | | $k_{10}^{\rm in}$=0.1 | a |
| (IIa,ATIII) | $E_2$ | $E_2,^{in}$ | | $k_2^{\rm in}$=0.2 | a |
| Binding (-,with-) | | | | | |
| (TFPI,Xa) | $TFPI$,$E_{10}$ | $TFPIa$ | $k^+_{tfpia:e10}=1.6 \cdot 10^7$ | $k^-_{tfpia:e10}=3.3 \cdot 10^{-4}$ | b |
| ($TFPIa$,TF:VIIa) | $TFPIa$,$E_7^{se}$ | $TFPIa : E_7^{se}$ | $k^+_{tfpia:e7se}=1.0 \cdot 10^7$ | $k^-_{tfpia:e7se}=1.1 \cdot 10^{-3}$ | b |

Table 7: INHIBITION REACTIONS (a) We estimate these parameters based on the half-lives of Factors IXa, Xa, IIa in plasma (Rosenberg & Bauer 1994). (b) From (Jesty et al. 1994). APC is not included in these simulations.

| Transition | Initial State | Final State | $M^{-1}sec^{-1}$ | $sec^{-1}$ | Note |
|---|---|---|---|---|---|
| | | | | | |
| Unactivated platelet adhering to SE | $P^{m,u}$ | $P^{se,a}$ | $k_{adh}$=2 $\cdot 10^{10}$ | | a |
| Bound platelet adhering to SE | $P^{b,a}$ | $P^{se,a}$ | $k_{adh}$=2 $\cdot 10^{10}$ | | a |
| Activated platelet adhering to SE | $P^{m,u}$ | $P^{se,a}$ | $k_{adh}$=2 $\cdot 10^{10}$ | | a |
| Activated platelet cohering to bound platelet | $P^{m,a}$ | $P^{b,a}$ | | $k_{coh} \cdot P_{max} = 1 \cdot 10^4$ | |
| Platelet activation by ADP | $P^{m,u}$ | $P^{m,a}$ | | $k_{adp}^{pla}$=0.34 | b |
| Platelet activation by thrombin | $P^{m,u}$ | $P^{m,a}$ | | $k_{e_2}^{pla}$=0.50 | b |

Table 8: PLATELET TRANSITIONS (a) Estimated from data in (Turitto & Baumgartner 1979, Turitto et al. 1980) as described in (Kuharsky & Fogelson 2001). (b) Estimated from data in (Gear 1994) as described in text.

# References

Ahmad, S. S., Rawala-Sheikh, R. & Walsh, P. N. (1989), 'Comparative interactions of Factor IX and Factor IXa with human platelets', *J. Biol. Chem.* **264**, 3244–3251.

Brass, L., Ahuja, M., Belmonte, E., S, S. P., Tarver, A. & Hoxie, J. (1994), 'The human platelet thrombin receptor. Turning it on and turning it off.', *Ann. N. Y. Acad. Sci.* **714**, 1–12.

Butenas, S. & Mann, K. G. (1996), 'Kinetics of human Factor VII activation', *Biochemistry* **35**, 1904–1910.

Gear, A. R. L. (1994), 'Platelet adhesion, shape change, and aggregation: rapid initiation and signal transduction events', *Can. J. Physiol. Pharmacol.* **72**, 285–94.

Grabowski, E., Franta, J. & Didisheim, P. (1978), 'Platelet aggregation in flowing blood *in vitro* II. Dependence of aggregate growth rate on ADP concentration and shear rate', *Microvasc. Res.* **16**, 183–195.

Hill-Eubanks, D. C. & Lollar, P. (1990), 'von Willibrand factor is a cofactor for thrombin-catalyzed cleavage of the Factor VIII light chain', *J. Biol. Chem.* **265**, 17854–8.

Jesty, J., Wun, T. & Lorenz, A. (1994), 'Kinetics of the inhibition of Factor Xa and the Tissue Factor-Factor VIIa complex by the Tissue Factor Pathway Inhibitor in the presence and absence of heparin', *Biochemistry* **33**, 12686–12694.

Krishnaswamy, S., Jones, K. C. & Mann, K. G. (1988), 'Prothrombinase complex assembly. Kinetic mechanism of enzyme assembly on phospholipid vesicles', *J. Biol. Chem.* **263**, 3823–3834.

Kuharsky, A. & Fogelson, A. (2001), 'Surface-mediated control of blood coagulation: The role of binding site densities and platelet deposition', *Biophys J* **80**, 1050–1074.

Leiderman, K. & Fogelson, A. (2011), 'Grow with the flow: a spatial-temporal model of platelet deposition and blood coagulation under flow', *Math Med Biol* **28**, 47–84.

Leiderman, K., Miller, L. & Fogelson, A. (2008), 'The effects of spatial inhomogeneities on flow through the endothelial surface layer', *J Theor Biol* **252**, 313–325.

LeVeque, R. J. (1996), 'High-resolution conservative algorithms for advection in incompressible flow', *SIAM J. Numer. Anal.* **33**, 627–665.

Lollar, P., Knutson, G. J. & Fass, D. N. (1985), 'Activation of porcine Factor VIII:C by thrombin and Factor Xa', *Biochemistry* **24**, 8056–8064.

Mann, K. G. (1987), 'The assembly of blood clotting complexes on membranes', *TIBS* **12**, 229–233.

Mann, K. G. (1994), Prothrombin and thrombin, *in* R. Colman, J. Hirsh, V. Marder & E. Salzman, eds, 'Hemostasis and Thrombosis: Basic Principles and Clinical Practice', 3d edn, J.B. Lippincott Company, Philadelphia, PA, pp. 184–199.

Mann, K. G., Bovill, E. G. & Krishnaswamy, S. (1991), 'Surface-dependent reactions in the propagation phase of blood coagulation.', *Ann. N. Y. Acad. Sci.* **614**, 63–75.

Mann, K. G., Krishnaswamy, S. & Lawson, J. H. (1992), 'Surface-dependent hemostasis', *Semin. Hematol.* **29**, 213–26.

Mann, K. G., Nesheim, M. E., Church, W. R., Haley, P. & Krishnaswamy, S. (1990), 'Surface-dependent reactions of the vitamin K-dependent enzyme complexes', *Blood* **76**, 1–16.

Monkovic, D. D. & Tracy, P. B. (1990*a*), 'Activation of human Factor V by Factor Xa and thrombin', *Biochemistry* **29**, 1118.

Monkovic, D. D. & Tracy, P. B. (1990*b*), 'Functional characterization of human platelet-released Factor V and its activation by Factor Xa and thrombin,', *J. Biol. Chem.* **265**, 17132–40.

Morrissey, J. H. (1995), 'Tissue Factor modulation of Factor VIIa activity: Use in measuring trace levels of Factor VIIa in plasma,', *Thromb. Haemost.* **74**, 185–188.

Nemerson, Y. (1992), 'The Tissue Factor pathway of blood coagulation', *Semin. Hematol.* **29**, 170–176.

Nesheim, M. E., Pittman, D. D., Wang, J. H., Slonosky, D., Giles, A. R. & Kaufman, R. J. (1988), 'The binding of s-labeled recombinant Factor VIII to activated and unactivated human platelets', *J. Biol. Chem.* **263**, 16467.

Nesheim, M. E., Tracy, R. P., Tracy, P. B., Boskovic, D. S. & Mann, K. G. (1992), 'Mathematical simulation of prothrominase', *Methods Enzymol.* **215**, 316–328.

Novotny, W. F., Brown, S., Miletich, J., Rader, D. & Broze, G. (1991), 'Plasma antigen levels of the lipoprotein-associated coagulation inhibitor in patient samples', *Blood* **78**, 387–93.

Rawala-Sheikh, R., Ahmad, S. S., Ashby, B. & Walsh, P. N. (1990), 'Kinetics of coagulation Factor X activation by platelet-bound Factor IXa', *Biochemistry* **29**, 2606–11.

Rosenberg, R. & Bauer, K. (1994), The heparin-antithrombin system: A natural anticoagulant mechanism, *in* R. W. Colman, J. Hirsh, V. J. Marder & E. W. Salzman, eds, 'Hemostasis and Thrombosis: Basic Principles and Clinical Practice', 3d edn, J.B. Lippincott Company, Philadelphia, PA, pp. 837–860.

Turitto, V. T. & Baumgartner, H. R. (1979), 'Platelet interaction with subendothelium in flowing rabbit blood: Effect of blood shear rate', *Microvasc. Res.* **17**, 38–54.

Turitto, V. T. & Leonard, E. F. (1972), 'Platelet adhesion to a spinning surface', *Trans. Amer. Soc. Artif. Int. Organs* **18**, 348–54.

Turitto, V. T., Weiss, H. J. & Baumgartner, H. R. (1980), 'The effect of shear rate on platelet interaction with subendothelium exposed to citrated human blood', *Microvasc. Res.* **19**, 352–365.

Walsh, P. N. (1994), Platelet-coagulant protein interactions, *in* R. W. Colman, J. Hirsh, V. J. Marder & E. W. Salzman, eds, 'Hemostasis and Thrombosis: Basic Principles and Clinical Practice', 3d edn, J.B. Lippincott Company, Philadelphia, PA, pp. 629–651.

Weiss, H. J. (1975), 'Platelet physiology and abnormalities of platelet function (Part 1)', *New Engl. J. Med.* **293**, 531–541.

Young, M., Carroad, P. & Bell, R. (1980), 'Estimation of diffusion coefficients of proteins', *Biotech. and Bioeng.* **22**(5), 947–955.
